# Do political parties matter for property taxes?

**DOI:** 10.1371/journal.pone.0319994

**Published:** 2025-05-22

**Authors:** Felipe Aldunate, Cristobal Diaz, Santiago Truffa

**Affiliations:** 1 ESE Business School, Universidad de los Andes, Santiago, Chile; 2 Pontificia Universidad Catolica de Chile, Santiago, Chile; Federal University of ABC, BRAZIL

## Abstract

We evaluate whether political partisanship affects local taxes in an emerging economy. Using detailed residential property-level data in Chile, we study whether mayors’ political leanings affect the reassessment process and thus the taxes paid by home owners. In Chile, this type of tax is especially relevant since it is one of the largest sources of municipal income. To address endogeneity concerns, we use a regression discontinuity design, exploiting the quasi-experimental variation provided by close municipal elections. Our main results show that after a right-wing mayor is elected, property assessments increase up to 31% more than in a similar municipality where a left-wing mayor was elected. This effect cannot be fully explained by changes in prices or property characteristics.

## Introduction

Many local governments in developing countries face the near impossible task of funding the infrastructure and services required to meet the basic needs of growing urban populations, a challenge that has been recently highlighted by social unrest in several countries including Bolivia, Chile, Colombia, and Ecuador.

To boost revenue and meet the demands of their constituents, local politicians can utilize property taxes. Often, homeowners with properties valued below a certain threshold are exempt from this tax. Consequently, politicians’ motivations to modify property taxes may be influenced by their political ideologies, balancing differing views on redistribution with the necessity of funding local infrastructure.

In this paper we evaluate whether political factors affect local property taxes in an emerging economy: Chile. Specifically, we study whether a mayor’s political leanings affects the reassessment process and thus the taxes paid by home owners. For several reasons Chile is particularly interesting to study this question. First, local property taxes constitute a significant portion of municipal income (around 32%), with the associated sharing rules enabling the funding of essential services in poorer municipalities. Second, major reassessments occur simultaneously for all properties every four year, the same frequency as the local political election cycle. There is a two-year delay between the reassessment cycle and political elections, which gives us a reasonable amount of time to see any effects materialize. This institutional feature allows us to contrast reassessment outcomes with the outcomes of local political elections. Third, the developing country context with a weaker institutional environment provides local politicians with potential maneuvering space to strategically influence the reassessment process, a dynamic less observable in developed economies. Finally, we have access to rich micro-level property data, enabling rigorous econometric analysis and causal inference techniques.

According to the Chilean Internal Revenue Service (Servicio de Impuestos Internos or SII), these processes consider variables such as the behavior of the real estate market, road infrastructure, urban equipment, public investment, building category, and land use. In principle, this process should be independent of partisan politics. However, politicians’ incentives to influence local tax policy might be stronger in developing countries due to the greater unmet needs and demands of local populations. Additionally, weaker rule of law could enable local politicians to exert influence over property tax policies beyond their constitutional limits. In such weaker institutional environments, local political actors might strategically consider law enforcement as a factor in their decision-making process.

However, isolating the effect of political partisanship on local tax collections through property reassessments is not an easy task because of factors related to property reassessment such as spending or property values. To alleviate this endogeneity concern, we follow a regression discontinuity design approach, exploiting the quasi-experimental variation that occurs as a result of close municipal elections. In Chile, mayors are elected via direct voting, and the candidate with the most votes is elected. The idea is that municipalities where a right-wing candidate won by a small margin, are otherwise similar to the municipalities where a left-wing candidate won by a small margin.

We combine two different datasets for our main results. First, we use administrative data from the SII. The panel data provided by this institution includes yearly information between 2009 and early 2019 for all properties. The dataset contains detailed information about tax assessments and observable property characteristics. Second, we collect data on municipal elections from the Chilean Electoral Service (SERVEL). These elections are held simultaneously in all Chilean municipalities every four years. We collect data for 2012 and 2016, that is two years before each housing re-valuation process. The data include the percentage of votes for each candidate, which allows us to employ a regression discontinuity approach. We classify candidates on their political affiliation, including an “independent” category. Overall in our sample, 39% and 45% of elections are won by right- and left-wing candidates respectively (independent candidates win the remainder).

We use property characteristics data to perform a balance test before the elections between municipalities where a right-wing (left-wing) candidate was narrowly elected. We also collect data on the characteristics of elected politician and political candidates. This data allows us to further evaluate selection challenges, such as potential compensating differentials. Finally, we collect property-level commercial prices for all chilean municipalities. With this information we investigate whether an increase in commercial property values could explain the increase in property reassessments.

Our main results show that after a right-wing mayor is elected, residential property assessments increase up to 31% more than in a similar municipality where a left-wing mayor was elected. Interestingly the results are only statistically significant for the first reassessment process in our sample (2014), and not for the second one (2018). The 2014 results are robust to the kernel choice, higher-order polynomial, and the inclusion of observable characteristics at the property level, average prices and average incomes at the municipality level.

Finally, using the universe of property transactions in Chile, we provide evidence that only a small portion of the observed effect can be attributed to changes in commercial prices. Similarly, changes in property-level characteristics, which could indicate the operation of an enforcement mechanism, do not appear to play a significant role in driving our results either.

The rest of the paper is organized as follows. Sect 1.1 presents a literature review and discusses the theoretical framework. Sect 2 discusses potential mechanisms explaining our results. Sect 3.1 discusses the Chilean municipal tax system. A description of our data is presented in Sect 3.2. Sect 3.3 describes our empirical strategy. Sect 4 presents the main empirical analysis, and Sect 4.2 presents a series of robustness exercises. Sect 5 presents a discussion of the results and links them to the theoretical framework. Finally, Sect 6 concludes.

### 1.1 Literature review and theoretical framework

The influence of political factors on municipal financial management has been a general subject of interest [1–3]. Many authors have analyzed how political factors can predict specific aspects of financial management [4,5]. Yet, at the municipal level, few works have researched any aspect of public finances, and they have ambiguous results. For instance, [6] finds a robust effect of local government’s political characteristics on budget deficits, while [7] concludes that there is no long-term effect, but only a correlation in the short term between debt levels and the number of parties in a coalition.

There is little evidence regarding the effects of partisanship on property assessments. It has been shown that elected assessors are more sensitive to political influences [8]. Similarly, it has been documented that the political leaning of the mayor can influence land use regulation. Using data from Spanish cities during the 2003–2007 political term, it was found that cities controlled by left-wing parties converted significantly less land from rural to urban uses compared to those controlled by right-wing parties [9].

This paper elaborates on the property tax literature for developing countries. To our knowledge, it represents the first approach to study the influence of political factors on the indirect mechanisms that influence the determination of said tax in said countries. This is especially relevant because it is a progressive tax that allows more resources to be allocated to social spending at the expense of higher taxes for the wealthy. Therefore, politicians from different factions may have different preferences on the topic.

Our paper also relates to the literature examining the effects of party affiliation on local fiscal and tax policies. It has been shown through a regression discontinuity analysis for U.S. cities that the mayor’s party does not affect any of the studied policy outcomes, including the size of the city government and the allocation of public spending [10]. Evidence suggests that the mayor’s party does influence public safety spending but not tax or social policies in the U.S. [11]. It has been argued that this may be due to the overlap across local, state, and federal governments, which can limit the influence of local governments. Using Swedish local government data and a regression discontinuity (RD) analysis, it has been concluded that left-wing governments impose a 2%–3% higher taxes compared to right-wing governments [12].

It has been concluded that party representation does not affect tax policy in Swedish municipalities [13]. Ambiguous results have been found for different left-wing parties in Germany [14], while increased property taxes have been observed in Norwegian municipalities when left-wing parties hold more power [15]. Notably, these studies focus primarily on the developed world. This paper complements the existing literature by examining the effects of political partisanship on local taxes in an emerging economy, where, as previously explained, the incentives for politicians to increase revenue may be stronger. While some research has explored developing nations, such as the study on political partisanship and local fiscal policy in Brazil [16], local tax policy has not been the focus of these analyses, which is the primary contribution of our study.

Our paper also relates to a broader literature that examines these questions at the state and national levels. It has been concluded that there are few differences in 32 policy settings and economic outcomes under Democratic and Republican governors in the U.S., with no observed effects of political partisanship on state tax rates [17]. Using a panel of U.S. states, it has been found that the impact of political partisanship on state tax policy depends on the governor’s eligibility for re-election. Specifically, Democrats tend to increase income taxes relative to Republicans when they are “re-electable”, but they reduce taxes when they are not [18]. Similarly, it has been shown that the governor’s party does not affect total spending but influences the allocation of funds [19]. Consistent with these findings, we show that factors such as whether mayors are re-elected and their political alignment with the central government are key mediators of our effects, even in underdeveloped countries.

This paper contributes to the economic literature on the political economy of taxation by providing empirical evidence on how local political dynamics affect property tax reassessments. It has been emphasized in existing works, such as [20], that state capacity plays a critical role in shaping taxation outcomes, with institutional quality and governance being key factors. Additionally, the challenges of property taxation outlined by [21] are expanded upon in this study, which demonstrates how political partisanship introduces inefficiencies and biases into the reassessment process. Furthermore, this research aligns with [22], who highlights the importance of enforcement in shaping tax compliance and efficiency, by examining enforcement as a mechanism through which political actors influence fiscal outcomes.

Lastly, this paper contributes to the literature on clientelism discussed by [23], which examines how the exchange of material goods for electoral support can lead to reduced welfare program coverage, increased corruption, and weakened rule of law. Building on this framework, our study investigates whether local politicians in Chile use property tax reassessments as a tool for clientelistic practices. The findings indicate that political leanings significantly impact tax policies, with right-wing mayors being more likely to increase property assessments, potentially utilizing the resulting revenues for public goods provision.

The impact of anti-property-tax evasion programs on electoral outcomes has been investigated, showing that voters respond to tax enforcement policies [24]. The responsiveness of officials to voter preferences for property taxes in New York State towns has also been examined, comparing outcomes when tax assessors are either appointed or elected by popular vote [25]. While Sances’ work explores the dynamics of elected versus appointed officials in the U.S., our study expands this discussion by focusing on how political partisanship influences the behavior of appointed officials in Chile. By examining property tax assessments in a centralized system, we provide new insights into the interplay between local political dynamics and tax administration.

This paper relates closely to [26] by examining the intersection of political incentives and taxation. Similarly, [27] explore how property taxation intersects with fiscal infrastructure and electoral incentives in Brazil. They study how property titling facilitates tax collection and encourages private investment in Brazilian municipalities, showing that property tax revenue increases significantly following cadastre updates. Our research examines how political orientation affects incentives to update the cadastre and enhance fiscal capacity. Furthermore, our study extends this framework to Chile, demonstrating that even within a centralized tax collection system, local political dynamics can shape property reassessment outcomes.

Finally, this paper contributes to the political science literature on the intersection of taxation and governance, particularly in emerging markets. The concept of forbearance, where political actors strategically choose not to enforce certain laws to gain political leverage, has been explored by [28]. This discussion is expanded in our findings, which illustrate how enforcement can also shape property tax reassessments, serving as a tool for political influence. Additionally, the argument by [29] that taxation fosters accountability and responsiveness is extended by showing how political partisanship may undermine the neutrality of tax systems, particularly in centralized tax collection frameworks. Evidence from an emerging market is also provided, contributing to the growing body of work on the role of partisan alignment in local governance (e.g., [11,12]), and demonstrating how local political actors can influence fiscal outcomes despite having limited formal control over tax collection.

In summary, the literature studying the effects of political partisanship on fiscal policies, specifically on tax policy, has inconclusive results. Our main contribution to the literature is that, to the best of our knowledge, we are the first paper to present an RD design to explore local property taxes in an emerging economy.

## 2 Potential mechanisms

After a right-wing mayor is elected, we observe an increase in property reassessments. This finding is somewhat unexpected, as previous political science literature suggests that left-wing politicians are typically more focused on raising and collecting taxes for redistribution to their lower-income constituents [30–32]. We propose two alternative explanations for our main finding: (1) a price effect due to the right-wing mayor’s greater ability to produce public goods, and (2) stricter enforcement of property reassessment laws by right-wing mayors compared to their left-wing counterparts.

The pricing channel suggests that the commercial value of properties increases due to better municipal management under right-wing mayors. This hypothesis posits that a right-leaning administration enhances the district’s value, leading to higher commercial property values and prices. However, this idea is controversial and somewhat improbable for several reasons. First, the time frames between reassessments seem too short for a municipal administration to produce significantly more quality public goods and for these improvements to impact property prices. Public infrastructure investments take years to complete and to influence real estate prices. Additionally, it would require that right-wing mayors have superior management capabilities to deliver this added value. While this is debatable, it remains an empirical question. We examined the universe of municipalities in Chile and found that local prices do not fully account for the effects we observed.

Another possibility is the differential willingness to enforce the law. This is particularly relevant in Chile, where it is common to make home improvements and expansions that must be declared to the municipality for legal recognition. These formalities are costly, and regularization means higher taxes for the properties. Regularization costs are significant, as hiring an architect is usually required to get an official municipal reception for a property. Since 2023, a special law known as *La Ley del Mono* has allowed for expansion permits with just a sketch or drawing of the project, aiming to encourage property owners to regularize their properties and pay the associated taxes.

Mayors who want to collect more taxes can enforce different measures to control informal property expansions. Others might choose not to enforce these measures, practicing forbearance [28]. Forbearance describes the intentional and irrevocable non-enforcement of laws by politicians to maximize votes and rents. This strategic non-enforcement is prevalent in contexts where resource constraints or bureaucratic inadequacies hinder effective law enforcement. Holland’s work focuses on urban Latin America, demonstrating how politicians use forbearance to manage street vending and squatting laws, signaling distributive commitments to mobilize voters. Unlike situations where laws are not enforced due to a lack of state capacity, forbearance requires a political decision not to enforce a law, hinging on the discretion politicians have over enforcement.

The forbearance hypothesis serves as a plausible explanation in the case of property reassessment because first, it can be revoked, allowing politicians to retain the threat of enforcement as leverage. Second, it can be hidden from legislative debate and oversight, enabling discretion in its application. Third, it allows for the targeting of resources to those willing to bear the costs of illegality.

In Sect 5 we test whether the non-enforcement of the law can be a driving mechanism behind our results. To do so, we explore whether the political ideology of the municipal mayor affects variables such as the official property characteristics (such as built surface).

### 2.1 Variables affecting appraisals

In theory, given the model presented in S1 Appendix, property appraisals should only be affected by the factors included in the appraisal process. By law, the SII has to be transparent about the variables considered. Thus, theoretically, the analysis can be replicated and would find the same appraisals. It is important to note that we are not using the model in any way to compute the dependent variable of our study, as we are using the official values reported by the SII. How these parameters are determined [33], however, is not entirely transparent. The SII argues that it is based on the behavior of the real estate market, road infrastructure, urban equipment, public investment, building category, and land use. The sources used to establish these criteria are not clear, and as reported by [34], there may be an “arbitrary discrimination" towards certain types of properties.

As already mentioned, although municipalities should not be able to directly affect the assessment’s parameters, they are in charge of providing the necessary information used in the assessment. One problem is that this is an expensive process, and, as [35] suggests, it can entail both administrative and political costs since people could negatively associate tax increases with the municipal authorities. On the other hand, an increase in property taxes would increase the municipal budget. Indeed, in S1 Appendix we explain the rule for how property tax payments are split between municipalities.

These ambiguities in the process raise questions on whether systematic elements may be influencing decision-making. As the literature [14] shows, there is evidence of a desire on the part of municipal authorities to affect property taxes. Chilean municipalities could indirectly affect these taxes through the information provided to the SII. On the other hand, as reported by [9], it is also possible that political partisanship affects the urban development policies taken on by local governments. Hence, political partisanship could correlate with assessments through commercial prices.

## 3 Material and methods

### 3.1 Property appraisals and taxes in Chile

The basis for the current Chilean property tax was established in 1998 by [36]. Although its exact operation has undergone subsequent modifications [37], the law establishes a property tax levied based on the appraisal of property value, determined by the provisions of the code. It also established that the Chilean revenue collection agency (*Servicio de Impuestos Internos or SII*) is the institution that must carry out the appraisal process and reassess agricultural and non-agricultural real estate every four years. Importantly, the SII can request the assistance and cooperation of the municipalities in the appraisal process.

The law also establishes that not all properties are subject to taxation. In the case of non-agricultural properties with a residential destination, after the last reassessment process in 2018, properties whose assessed value was less than USD 44,865 were exempt (throughout this article, we use an exchange rate of 740 Chilean pesos per 1 US dollar, the exchange rate at the end of December 2019). This is around 13.6% higher than the average assessed value of USD 39,499. There is also a reduced payment tranche, with a reduced rate is applied. After the last reassessment process, owners of only 23% of homes throughout Chile paid property taxes.

Once the appraisal is determined, the property tax is calculated for a property. Legally, total national collection cannot increase more than 10% from the reassessment. Hence, authorities must adjust contribution rates accordingly. Table 1 presents the standards established in the original law and used in the last reassessment processes for non-agricultural residential properties. For more details about the appraisal system, along with exceptional cases and fees that apply, see S1 Appendix.

**Table 1:** Property tax rates.

Tax rate	Law 17,235	2014	2018
Agricultural	1.0%	1.0%	1.0%
Non-agricultural	1.4%	1.2%	1.088%
Non-agricultural, residential (reduced rate tranche)	1.2%	0.98%	0.933%
Non-agricultural, residential	1.4%	1.143%	1.088%

This table shows the various tax rates for each type of property. Law 17,235 established the rate first in 1969. The 2014 and 2018 rates correspond to those established in each residential property reassessment process. Tax rates are slightly lowered with each reassessment to comply with the rule that the total national tax collection cannot increase more than 10% in a single year. *Source*: *Servicio de Impuestos Internos* (SII).

It is important to note that a significant portion of the property taxes levied goes directly to the municipality, and an additional percent returns to the municipality through a distributive mechanism called the Municipal Common Fund (*Fondo Común Municipal* or FCM), of which 57.5% of its funds comes from property taxes. This system is designed to allocate resources from wealthier municipalities to the poorer ones, in order to lower inequality, and guarantee a steady supply of public goods to all citizens. The FCM represents the primary source of income for the municipalities (an average 34% of their total income), and its funds come from different types of taxes. A quarter of the FCM is divided into equals parts for every municipality. The rest is redistributed through a system that takes into account factors such as the poverty rate and the number of non-taxed properties. In the case of property taxes, 40% stays in the municipality, while 60% goes to the FCM.

### 3.2 Data description

To study the effects of political partisanship on property taxes, we use data on the fiscal valuations and municipal elections for our main results. To complement these results we additionally include a limited sample of commercial prices and other municipal level data.

#### Fiscal valuations.

Fiscal valuations come from the SII’s official databases. This data set features panel data on all properties nationwide from 2009 to early 2019, which includes the tax assessment, the payments made, and the percentage of non-taxed properties. It also provides information on the property’s observable characteristics, such as the surface area by soil type and the surface area per construction type, together with its material, quality, age, and unique condition. The panel is not balanced as some properties exit the sample, and others enter over time.

The calculation for land appraisal involves factors like property size, land value in homogeneous areas, and various corrective coefficients. Construction appraisal is computed by summing up the values of individual structural segments, adjusted for specific conditions. Shared amenities appraisal is determined by the total appraisal value divided according to co-ownership agreements.

We do not use data from all years in the sample because valuations in a non-reappraisal year remain relatively constant, and the prices adjust only for inflation. Thus, we focus only on changes related to reassessment processes. There are two residential property reassessments in our sample: 2014 and 2018. We also drop all observations for which we have changes in observable characteristics such as remodeling or increases in the square footage. We do so to filter out changes in valuations related to differences in the property characteristics and not necessarily to the mayor.

Panel A in Table 2 presents descriptive statistics of these two reassessment processes, specifically the essential differences between the 2014 and 2018 ones. The 2018 process involved significant changes in fiscal valuations. Panel B of this table shows that these changes are not constant across pre-reassessments valuation deciles. This is important because a significant percentage of houses are not subject to property taxes.

**Table 2:** Appraisal changes by reassessment year

Panel A: Summary statistics					
Process	N	Average	Std. Dev.	Min	Max
2014 reassessment	4,332,542	0.041	0.275	-0.415	1.409
2018 reassessment	4,747,412	0.681	0.493	-0.135	2.977
**Panel B: Change in appraisal per decile of appraisal**					
**Decile**		**Average 2014 reassessment**		**Average 2018 reassessment**	
1		0.119		1.138	
2		0.034		0.944	
3		-0.001		0.858	
4		0.001		0.812	
5		0.010		0.698	
6		0.027		0.627	
7		0.026		0.559	
8		0.032		0.467	
9		0.068		0.365	
10		0.098		0.342	
N		4,332,542		4,747,412	

Appraisal changes are constructed using the tax assesment value before and after a reassessment and calculating the percentage increase. The data per year are trimmed at 1%. Deciles are constructed using the pre-reassessment appraisal value. *Source*: Constructed using data from *Servicio de Impuestos Internos* (SII).

It is important to note that the change in fiscal valuation is limited by -1, but it has no upper limit for assessment increases. However, extreme changes were excluded. Therefore we trim our sample at the 1% level.

#### Municipal elections and income.

The data on municipal elections comes from the Chilean Electoral Service (*SERVEL*). Since we are focused on 2014 and 2018, we use data from the 2012 and 2016 municipal elections. To classify the political orientation of each mayor, we follow the methodology proposed in [38]. This criterion consists of defining the candidates according to the coalition to which they belong, and classifying these coalitions to left and right according to whether they belong to parties traditionally associated with each political orientation.In particular, right wing parties are: the Independent Democratic Union (UDI) and National Renewal (RN); meanwhile left wing parties are: Christian Democracy (DC), Party for Democracy (PPD), Radical Party, Socialist Party (PS), and Communist Party (PC). If the candidate does not belong to any right or left-wing coalition, then she is defined as an “independent". The results of this classification are shown in Panel A in Table 3. Panel B shows that changes in political orientation are relatively common. In 38% of cases, the mayor comes from a different political orientation than the existing one.

**Table 3:** Election summary statistics

Panel A: Mayors by ideology by election year				
Election year	Right-wing	Left-wing	Independent	Total
2012	121	167	57	345
2016	145	141	59	345
**Panel B: Election results by political incumbency (mayors)**				
**Election year**	**Incumbent coalition**		**Different coalition**	**Total**
2012	198		147	345
2016	231		114	345
Total	429		261	690
Percentage	62%		38%	

Panel A shows mayoral elections for the various municipalities. A candidate is classified as *right-wing* or *left-wing* if the candidate identifies with a coalition that includes a traditionally right-wing or left-wing party. If the candidate does not fall into either of these categories, he or she is classified as an *independent*. Panel B shows the percentage of candidates who belonged to the same coalition as the previous mayor. If the mayor is of the same coalition as the former mayor, he or she is classified as belonging to the incumbent coalition. *Source*: *Servicio Electoral de Chile* (SERVEL).

A key element of our identification strategy is the margin by which the elected mayor won the election. In Chile, mayors are elected via direct voting, and the candidate with the most votes is elected. Therefore we group ballots at the candidate level and define the voting margin as the difference between the two most popular candidates (as margin from the right). When an elected candidate obtains a voting margin of α%, the variable is defined as α for the winning ideology and −α for his closest competitor. In all specifications this variable is centered at zero, and it is defined as the margin from the right every time a right wing candidate is included in the estimation (right margin and restricted margin results). However, when the top two candidates are a left-wing individual and an independent one, we have to redefine the margin as the difference between the voting percentage of the left wing candidate minus the independent one.

Mayors play a crucial role in Chile, as they are responsible for providing various local public goods. Specifically, municipalities manage public spaces such as parks and streets, and are in charge of street lighting. Additionally, they handle security issues, including local guards who help protect communities. Local public schools and health services also depend on municipalities. For these reasons, mayors are significant figures in local politics. Many have eventually featured as presidential candidates following successful mayoral terms.

For robustness, we consider a new specification of the margin variable, defined as *restricted sample margin*, which only considers cases in which the first and second place in the election include both a right-wing candidate and a left-wing candidate. This ensures that all municipalities have a defined political position. A restricted sample margin greater than 0 corresponds to a right-wing mayor being elected, while a negative value is a left-wing one. Table 4 shows the result of all the assignments described. The number of observations decreases because of these conditions. Restricting the sample to elections where the two top candidates were from the right and left coalitions only could potentially introduce selection bias in the results. If that were the case we could find different results between the right-margin and restricted-margin specifications. However as we will show in the results section of this paper, this will not be the case.

**Table 4:** Election margins of mayoral candidates by coalition

2012 election					
Margin	Obs.	Average	Std. Dev.	Min	Max
Right	272	-0.030	0.230	-0.817	0.613
Left	290	0.038	0.221	-0.613	0.817
Independent	92	-0.030	0.198	-0.611	0.433
Restricted sample	235	0.040	0.231	-0.817	0.613
**2016 election**					
**Margin**	**Obs.**	**Average.**	**Std. Dev.**	**Min**	**Max**
Right	263	0.014	0.253	-0.707	0.643
Left	286	0.002	0.266	-0.643	0.752
Independent	105	-0.041	0.246	-0.752	0.563
Restricted sample	222	0.011	0.263	-0.707	0.643

This table shows the summary statistics for the election margins. We define an election margin as the difference in votes between the winning candidate and the runner-up, even if the race included than two candidates. This means that coalitions are not present in every municipal election in the data, as the observations only take into account the top two candidates. A margin higher than 0% means this candidate was elected. *Source*: Constructed using data from *Servicio Electoral de Chile* (SERVEL).

#### Additional data.

To complement our main results, we use two additional datasets. First, we collect data from the Unemployment Insurance Database which includes information about all dependent workers over 18 years-old whose contracts are determined by the Labor code. We excludes apprentices, workers younger than 18 years old, domestic workers, the independent or self-employed, public sector officials, and retirees. We compute the average of workers’ taxable income at the semester-municipality level. This information is available for all municipalities in our sample and we use it as a control variable in our main specifications as explained below.

Second, to investigate the mechanisms driving our results, we leverage commercial property prices from the universe of transactions in Chile, drawn from administrative records provided by the SII. This dataset, which includes all transactions processed through notaries, allows us to construct a hedonic municipality-level price index.

Finally, we incorporated candidate-level characteristics for both elected officials and all candidates who participated in the elections under analysis. To achieve this, we manually collected data on each candidate’s birthdate and gender.

S1 Appendix presents a description of the different variables used.

### 3.3 Empirical framework

#### Identification strategy.

Estimating the influence of political factors on the fiscal appraisals is a challenging task since unobserved municipal characteristics could be correlated with the political orientation of the elected mayor. Thus traditional estimating methods could suffer from an endogeneity problem driven by omitted variables. To isolate the causal effect that a representative of a particular political orientation has on local policies, the political economy literature has exploited the quasi-experimental variation that occurs as a result of hard-fought elections. If we order the elections based on voting margins, then the elections closer to the cut-off point would resemble a random experiment, since the probability of having fallen to either side of the cutoff is the same. Formally, the analysis corresponds to a regression discontinuity design (RD).

The fundamental assumption for this analysis is that municipalities below the cutoff are similar to those above it. The only difference between them is the mayor’s political orientation. If an effect is found, we can establish a causal relationship between the changes in the tax appraisals and the mayor’s political ideology.

An essential element of the identification strategy is to determine which officials are responsible for implementing local policies. In this study, we focus on mayors as the main force of implementation. We do so because the mayor is the primary official in charge of the municipal administration, while the municipal council has only a supervisory and advisory role.

Finally, a more technical aspect of the identification strategy relates to the specifics of the appraisal changes. By law, the model’s parameters are updated jointly at the national level every four years in a reassessment process, with changes in other years restricted to adjust for inflation and resolve specific cases. Thus, we exploit the variation from national-level reassessments.

#### OLS model controlling for observable factors.

We start with an OLS model that includes observable property factors. A mentioned above, this approach is susceptible to an omitted variable problem. The estimated model is given by Eq 1.

$$\Delta Valuation_{ikt}=\alpha +\beta_{1}right_{kt}+\beta_{2}left_{kt}+\beta_{3}income_{kt}+\beta_{n}X_{it}+\epsilon_{ikt}$$ (1)

In the estimated model, $Valuation_{iky}$ corresponds to the price change for each property *i* in reassessment period *t* located in municipality *k*. The dummy variables *right*_*kt*_ and *left*_*kt*_ are equal to 1 if the mayors of municipality *k* was from a right or left-wing coalition respectively. Note that it is possible to include both variables because the omitted category is for independent mayors. *X*_*it*_ is a vector of control variables at the property-level *i*, including the land area, the area by type of material type and quality, the percentage of the property subject to different special conditions, and the average age of construction. We also control for *income*_*kt*_ the average income of workers in municipality *k* at the time of the reassessment process *t*.

#### Regression discontinuity design.

There could be unobservable municipality characteristics that influence the relationship between changes in fiscal appraisals and political elections. Thus our preferred specification corresponds to a model that filters out these effects by exploiting close elections. We use an RD that allows us to analyze the effect on the dependent variable at the discontinuity. The running variable corresponds to the margin obtained by the winning candidate. It is necessary to define a bandwidth interval around the cutoff to determine which observations are relevant to the analysis. For the choice of bandwidth, we implement the methodology proposed by [39], which establishes the criteria for choosing a robust optimal bandwidth, taking into account the bias that can occur by selecting large intervals (also known as the CCT criterion). The RD model is presented in Eq 2.

$$\Delta Valuation_{ikt}=\alpha +\beta_{1}Margin_{kt}+\beta_{2}Margin_{kt}D_{ikt}+\rho D_{ikt}+\epsilon_{ikt}$$ (2)

Where $Valuation_{iky}$ corresponds to the change in the price of each property *i*, for reassessment period *t*, located in municipality *k*. *Margin*_*kt*_ is a function of the voting margin, which does not necessarily follow a linear trend. Finally, *D*_*ikt*_ is a dummy variable, which is 1 if the observation falls to the right of the cutoff and 0 otherwise. Considering that our mayors classification consists of three categories (left, right, and independent), we estimate the model for each ideology separately and pool the other two groups. We then present the results from when restrict the sample to elections where only right and left-wing candidates were the top two candidates. The list of municipalities included in the RDD analysis (right and left margin) is presented for 2014 and for 2018 in the S1 Appendix.

We can estimate Eq 2 using polynomial or non-parametric methods such as linear regressions, following [40]. For the base case, we perform the estimation using first-degree (linear) polynomials. As a robustness exercise, we also estimate the model using quadratic and cubic polynomials. We then estimate the base model using a triangular kernel, and perform further robustness tests using Epanechnikov and uniform kernels.

Another issue to consider is the possible autocorrelation that may exist within spatial areas and how this can affect our standard errors. To account for this, we cluster the standard errors at the municipality level.

## 4 Main results

This section presents the main results of our study. We start with a balance test for different variables for a subsample of municipalities. Then we briefly discuss the OLS model and finally we present the main regression discontinuity results. Our preferred specification is the regression discontinuity design, because of the endogeneity concerns discussed above.

### 4.1 Baseline results

#### Municipalities’ characteristics.

Even though our empirical strategy is based on a regression discontinuity design to address the potential endogeneity between municipal characteristics and the mayor’s party, it is still interesting to explore potential differences between municipalities before the election. To do so, we collected a series of construction-related variables that we aggregate at the municipal level. These variables are the total construction surface, number of general constructions, and number of non-habitable constructions. A detailed description of the variables can be found on S1 Appendix.

Table 5 compares municipalities where a left-wing mayor was elected by a narrow margin to municipalities where a right-wing mayor was narrowly elected. Since we cannot use the same optimal bandwidth that we use in our main results because we do not have enough observations as information was not available for every municipality in the original sample, we select a 10% bandwidth to have enough observations to be able to perform this comparison. Panels A and B compare the variables in January 2012 and January 2016 respectively, that is, a few months before the October elections we exploit in our empirical design.

**Table 5:** Balance test for relevant variables before elections

2012 election							
	Left-wing			Right-wing			
Variable	Mean	Std. Dev.	Freq.	Mean	Std. Dev.	Freq.	p-value
Total general constructions surface	1,031,582	2,287,495	44	1,144,428	2,111,818	40	0.815
Number of general constructions	12,507	28,091	44	14,690	28,187	40	0.723
Number of non-habitable constructions	0.318	1.177	44	0.3	0.911	40	0.938
Average of workers’ income (clp)	371,898	96,702	44	373,188	125,957	40	0.958
**2016 election**							
	**Left-wing**			**Right-wing**			
**Variable**	**Mean**	**Std. Dev.**	**Freq.**	**Mean**	**Std. Dev.**	**Freq.**	**p-value**
Total general constructions surface	751,628	1,222,750	33	842,064	1,376,577	35	0.776
Number of general constructions	10,573	15,575	33	9,588	15,560	35	0.795
Number of non-habitable constructions	0.788	2.848	33	0.743	2.672	35	0.947
Average of workers’ income (clp)	492,591	103,536	33	542,939	199,164	35	0.200

This table shows the balance test for key variables on municipalities classified as either left-wing or right-wing before each election, which corresponds to the treatment under this approach. *Source*: Constructed using data from the Chilean Internal Revenue Service (*Servicio de Impuestos Internos or SII* and AFC.

Our analyses show that there are no statistically significant differences in any of the variables right before the 2012 elections as shown by the p-values in the last column of panel A in Table 5. If we analyze the economic significance of these differences, we note that the averages are similar, with the highest difference in percentage terms being 17% for the number of the general constructions variable. Overall, the average of the absolute differences of the variables is only 11% for 2012.

Finally, panel B of Table 5 presents the same comparison for January 2016, which is eight months before a municipal election. The results are similar to panel A. There are no variables with statistically significant differences between left-wing and right-wing municipalities. It is also important to note that the differences do not show a consistent pattern, suggesting that one type is not more or less disadvantaged than the other. For example, while municipalities where a left-wing candidate will narrowly win the election have a larger general construction surface, they also have a lower number of general constructions.

#### OLS model.

We start by briefly analyzing the 2014 reassessment process. As explained above, we restrict the sample to observations where the property characteristics have remained unchanged over the period. This restriction allows us to isolate the effects driven by variations in the land, building, or shared amenities that could affect property assessments. We present the OLS results in the S1 Appendix. In all columns, the dependent variable is the change in the assessment value at the property level.

Overall, the main conclusion of the OLS analysis is that while there seems to be a statistically significant association between partisanship and reassessments, there is little consistency in the estimates. In the S1 Appendix we also present estimations for regressions that pooling 2014 and 2018 data. The results are in line with by-year results discussed above.

#### Manipulation of elections.

**Fig 1:**
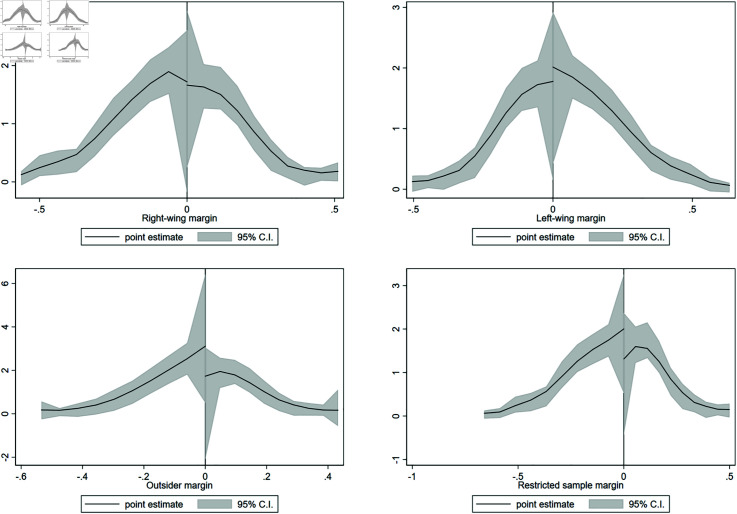
Density test: 2014 reassessment. This figure shows density estimators for the RD running variable, which is the margin obtained by the mayor of each coalition for the 2012 municipal election. A significant difference in the density near the cutoff would provide evidence of coalitions having a systematic impact on election results.

**Fig 2:**
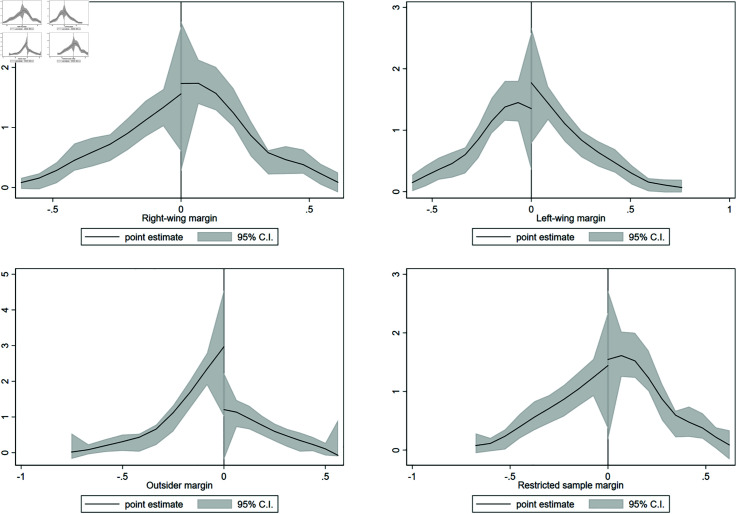
Density test: 2018 reassessment. This figure shows density estimators for the RD running variable, which is the margin obtained by the mayor of each coalition for the 2016 municipal election. A significant difference in the density near the cutoff would provide evidence of coalitions having a systematic impact on election results.

Before presenting the regression discontinuity analysis it is important to consider that when working with close elections, a possible concern is the presence of external influence on the running variable. To test this scenario in the discontinuity, we follow the methodology proposed by [41], which builds on [42]. As can be seen in Table 6, there is no evidence of manipulation on the variable that determines the treatment, that is, on the election margin. Fig 1 and Fig 2, show there is no significant difference in the density of observations on either side of the cut-off.

**Table 6:** Manipulation test applied to municipal elections

2012 election						
	Manipulation tests’ bandwidths		Effective N		Test	
	Left	Right	Left	Right	T	p-value
Right	0.187	0.171	85	77	0.383	0.700
Left	0.161	0.211	76	104	0.235	0.815
Independent	0.183	0.163	31	29	-1.208	0.227
Restricted sample	0.201	0.160	78	58	-0.713	0.458
**2016 election**						
	**Manipulation tests’ bandwidths**		**Effective N**		**Test**	
	**Left**	**Right**	**Left**	**Right**	**T**	**p-value**
Right	0.208	0.204	71	90	-0.135	0.893
Left	0.199	0.247	82	91	0.337	0.736
Independent	0.311	0.297	49	30	-0.984	0.325
Restricted sample	0.225	0.207	59	74	0.125	0.901

This table shows the manipulation tests results for the RD running variable, which is the margin obtained by the winning candidate. A p-value higher than 0.1 indicates that we cannot reject the null hypothesis that there is no discontinuity in the running variable at the cutoff, thus supporting the RD assumption of no manipulation. Each observation consists of a municipal election. *Effective N* corresponds to the number of observations that fall inside the manipulation tests’ bandwidths which are different that the optimal bandwidth in our preferred specifications. The RD coefficients are estimated using a triangular kernel. Significance levels: * *p*-value < .1, ** *p*-value < .05, *** *p*-value < .01.

#### Regression discontinuity analysis.

The previous section suggests a relationship between the mayor’s political orientation and changes in fiscal assessments and, thus, with municipal-level property taxes. However, in addition to the ambiguous results, there is a potential endogeneity problem related to omitted variables. In this section, we study whether there is a causal effect using a regression discontinuity design. This methodology allows us to have a good counterfactual that corrects for unobservable factors.

Table 7 presents the results when we pool the 2014 changes in appraisals with the ones in 2018. As mentioned above, both processes had many differences, but for completeness we start presenting them in one regression. We include year fixed effects in the estimation. Considering that the average change and its standard deviation were different between both processes, we normalize appraisal changes, dividing each value by the standard deviation of the changes of each process.

**Table 7:** Regression discontinuity results - Pooling 2014 and 2018

	Δ Appraisal	Bw	Eff. N	
			Left	Right
Right margin	0.237	0.164	1,513,757	1,860,511
	(0.270)			
Left margin	-0.356	0.128	1,600,869	1,227,549
	(0.309)			
Restricted sample margin	0.140	0.156	1,054,169	1,455,621
	(0.352)			

*Note*: This table shows RD estimates for changes in appraisals for both reassessment processes combined. We include year fixed effects to control for differences in each period. Each observation consists of a residential property and the dependent variable corresponds to the percentage change in its appraisal after each reassessment process normalized by the standard deviation of these changes in each process. *Effective N* corresponds to the number of observations that fall inside the optimal bandwidth. The restricted sample includes only observations in which the election was decided between a right-wing and left-wing candidate. The RD coefficients are estimated using a triangular kernel. Robust standard errors are clustered at the municipality level. Significance levels: * *p*-value < .1, ** *p*-value < .05, *** *p*-value < .01.

The results in Table 7 show that after a right-wing mayor is elected there is an increase in appraisal value equivalent to 0.24 standard deviations. Similarly, if a left-wing mayor is elected there is a 0.36 standard deviation decrease in appraisal value. Finally, the restricted sample, that keeps municipalities were the top two candidates were from the right or left leaning coalitions only, corroborate these results. However, none of these results are statistically significant.

Table 8 presents the main findings of this study. The results for 2014 and 2018 are presented in panels A and B respectively. The table shows that the effect is much higher than suggested by the OLS analyses, which highlights the importance of filtering out endogeneity concerns. The first row shows that a right-wing mayor’s election results in a 1.1 standard deviation increase in appraisal which is equivalent to a 31% additional increase in value. Similarly, if a left-wing mayor is elected, the assessment values decrease by 1 standard deviation. It is important to note that these are not considering the same sample because independent candidates are also included. The last row of panel A shows that if we restrict the sample to elections where the top two candidates are from either the right or the left, that is, we exclude the cases were an independent candidate was among the two top contenders, there is a similar effect associated with a victory by the right-wing candidate. These results are statistically and economically significant. Panel B of Table 8 present the same analysis for 2018 and shows that there is no longer an effect of political partisanship on reassessments in this second process. In S1 Appendix we show the same results but for a different choice of bandwidth. Specifically, we use the optimal bandwidth from the pooled RD for the by-year RD estimations. We find that this alternative specification does not change any of these conclusions.

**Table 8:** Regression discontinuity results - Optimal bandwidth in each estimation

**Panel A: 2014 reassessment**				
	**Δ Appraisal**	**Bw**	**Eff. N**	
			**Left**	**Right**
Right margin	1.140***	0.108	458,291	600,612
	(0.246)			
Left margin	-1.010***	0.097	660,316	523,098
	(0.256)			
Restricted sample margin	1.121***	0.117	467,769	660,338
	(0.244)			
**Panel B: 2018 reassessment**				
	Δ **Appraisal**	**Bw**	**Eff. N**	
			Left	Right
Right margin	-0.062	0.209	889,696	1,299,062
	(0.346)			
Left margin	0.195	0.234	1,285,621	922,385
	(0.368)			
Restricted sample margin	-0.482	0.196	678,951	783,656
	(0.521)			

*Note*: This table shows RD estimates for changes in appraisals for each reassessment process. Each observation consists of a residential property and the dependent variable corresponds to the percentage change in its appraisal after each reassessment process normalized by the standard deviation of these changes in each process. *Effective N* corresponds to the number of observations that fall inside the optimal bandwidth. The restricted sample includes only observations in which the election was decided between a right-wing and left-wing candidate. The RD coefficients are estimated using a triangular kernel. Robust standard errors are clustered at the municipality level. Significance levels: * *p*-value <.1, ** *p*-value <.05, *** *p*-value <.01.

**Fig 3:**
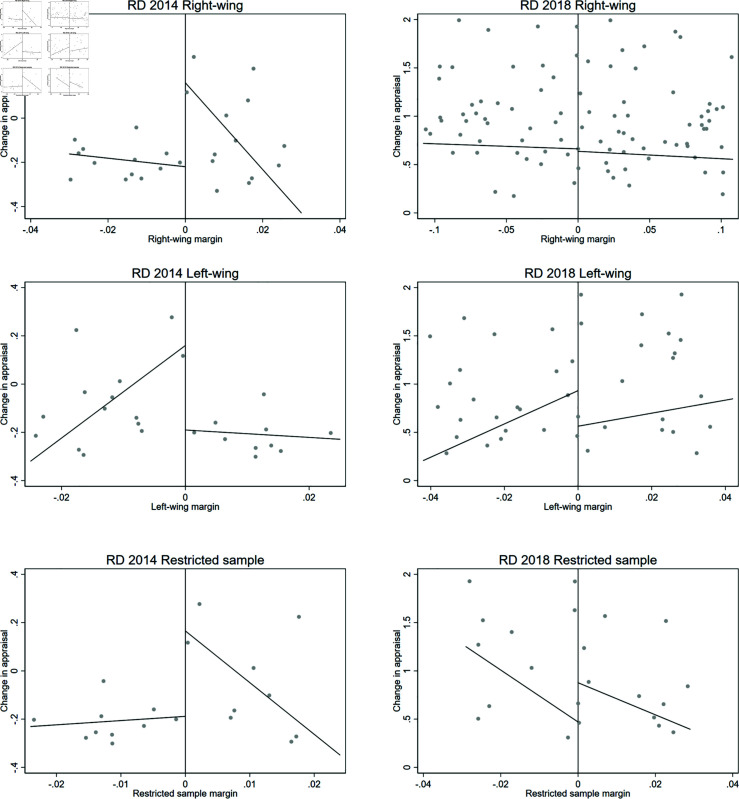
Regression discontinuity design using municipal elections. This figure shows regression discontinuity results for close elections (2012 and 2016 municipal elections) and their subsequent effect on property appraisals. The running variable is the margin between the top two candidates in the election, so plots on the right side of the cutoff are elected candidates. The bottom row only includes observations in which the election was decided between left-wing and right-wing candidates (i.e., the restricted sample). In this case, falling to the right of the cutoff means the right-wing candidate was elected, while falling to the left means the left-wing candidate was elected.

Fig 3 presents these results graphically. The change in the appraisals is plotted on the y-axis. The x-axis is the candidate’s victory margin. In the top two plots, a positive value in the x-axis corresponds to the margin by which a right-wing candidate won the election. The strong effect is corroborated in the top left figure, while the top right figure clarifies why there was no effect on Table 8 panel B for this specification. Next, the top middle graphs correspond to the margin of left-wing candidate victories over all other candidates. Here, we observe a significant jump at the cutoff in the figure for 2014, suggesting a negative effect on valuations after a left-wing candidate is elected. Finally, the bottom row shows the results for the restricted sample, where a positive value corresponds to a victory by the right-wing candidate. As explained above, the results are consistent with independent mayors behaving similarly to right-wing candidates in terms of policies that affect property appraisals.

Overall, our main results show that the mayor’s political partisanship affects reassessment outcomes. In our preferred specification, a right-wing candidate election over a left-wing one results in an increase in property valuation between up to 31%. This could imply that right-wing local politicians exert an effort to increase property taxes. Alternatively, this result could be driven by changes in commercial prices that drive increased valuations. We explore this alternative hypothesis in Sect 5.

### 4.2 Robustness

In this section, we present different robustness tests. We start with an analysis of compensating differentials as suggested by [43]. Next, we analyze the sensitivity of our results to different bandwidth specifications. We also discuss the effect of changing kernels and the order of the polynomial. Finally, we end the section with the results of a falsification test. Overall, our main results are robust to all of these alternative specifications.

**Compensating Differentials.** A key limitation of close-election regression discontinuity (RD) designs is that they often conflate the treatment effect of partisanship with compensating differentials stemming from individual politician characteristics, such as competence, charisma, or political networks [43]. This article underscores the need for caution in attributing observed effects solely to partisanship and calls for rigorous testing to determine whether these compensating differentials might contribute to or explain the results. In our case, the observed impacts of mayoral partisanship on property reassessments could partially reflect individual traits of the mayors, rather than purely their partisan affiliation. For instance, right-wing mayors might differ in their administrative skills or relationships with the central tax authority, which could amplify or dampen the effects attributed to partisanship.

To address these concerns, we manually collected data on all mayoral candidates for the 2012 and 2016 elections. Specifically, we gathered their birth dates to calculate age as a proxy for experience and we also recorded their gender. Using these variables, we tested for any discontinuity around the threshold. The underlying rationale is that if selection on unobservables exists, some of it might be captured by controlling for observable characteristics correlated with these unobservables.

**Fig 4:**
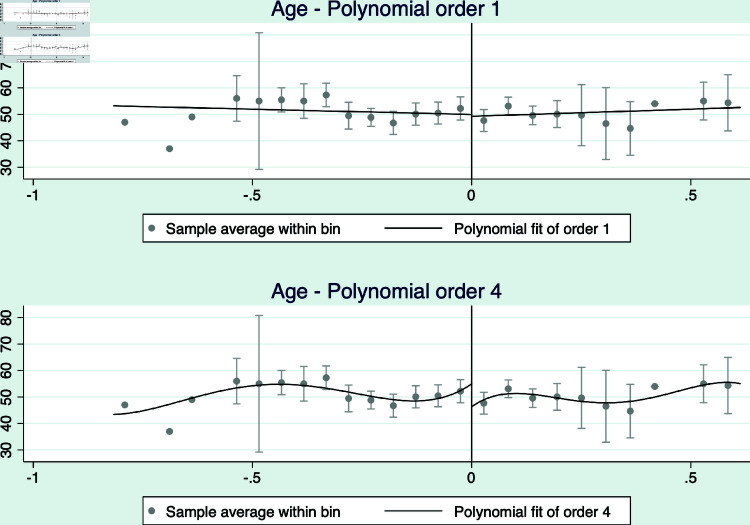
RD 2014 - Age of elected mayor. This figure shows density estimators for the RD running variable, which is the margin obtained by the mayor of each coalition for the 2012 municipal election. A significant difference in the density near the cutoff would provide evidence of coalitions having a systematic impact on election results.

**Fig 5:**
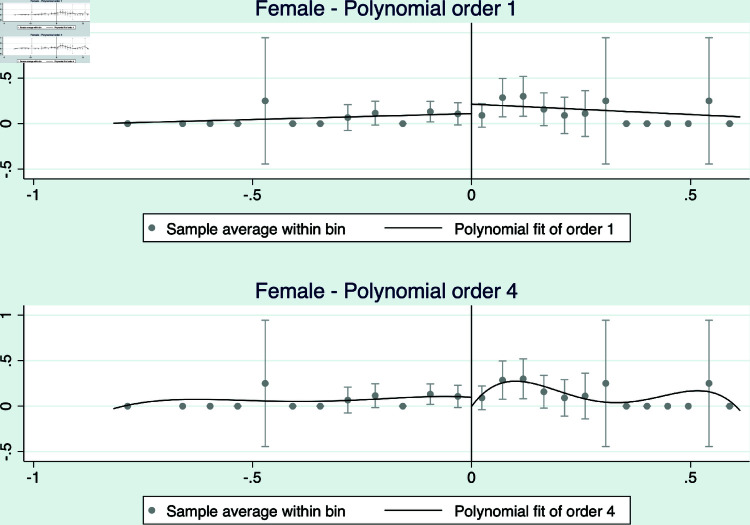
RD 2014 - Gender of elected mayor (female =1). This figure shows density estimators for the RD running variable, which is the margin obtained by the mayor of each coalition for the 2012 municipal election. A significant difference in the density near the cutoff would provide evidence of coalitions having a systematic impact on election results.

The results are presented in Figs 4 and 5, and Table 9. The figures presents the RD analyses using a linear and a quadratic polynomial for completeness. Note that we only show the estimations for the restricted margin analyses, which is our preferred specification as it restricts the sample to elections decided by a narrow margin between a right and left-wing candidate. All figures suggest that there are no differences between right and left wing elected mayors in terms of age, as a proxy for experience, or gender of the elected mayors.

**Table 9:** Regression discontinuity results for 2014 - Compensating differential analyses

**Panel A: Age of mayor**				
	**Age**	**Bw**	**Eff. N**	
			**Left**	**Right**
Right margin	-4.490	0.164	80	74
	(4.227)			
Left margin	5.081	0.145	70	78
	(1.243)			
Restricted sample margin	-5.233	0.157	64	57
	(4.446)			
**Panel B: Gender of mayor (female indicator)**				
	**Female**	**Bw**	**Eff. N**	
			**Left**	**Right**
Right margin	-0.057	0.120	63	55
	(0.131)			
Left margin	0.113	0.107	58	56
	(0.110)			
Restricted sample margin	-0.146	0.098	42	38
	(0.123)			

*Note*: This table shows RD estimates for the age and gender of elected mayors in the 2012 election. Each observation consists of a municipality. In panel A the dependent variable is the age of the elected candidate. In panel B the dependent variable is an indicator variable equal to 1 if the elected candidate is female. *Effective N* corresponds to the number of observations that fall inside the optimal bandwidth. The restricted sample includes only observations in which the election was decided between a right-wing and left-wing candidate. The RD coefficients are estimated using a triangular kernel. Significance levels: * *p*-value < .1, ** *p*-value < .05, *** *p*-value < .01.

Furthermore, Table 9 presents the estimation results for both variables for all specifications. The estimated coefficients and its standard errors support the evidence presented in the figures. Overall, the evidence allows us to conclude that there are no statistically significant differences between elected mayors in our RD analyses.

However, as [43] cautions, the absence of discontinuities in observables does not confirm the validity of a regression discontinuity (RD) design, as it could result from insufficient statistical power or unaccounted-for unobservable factors. Acknowledging these limitations, we interpret our findings with caution, recognizing that observable characteristics may not fully capture underlying biases. In our case, the observed effects could for example reflect a combination of partisan ideology and individual traits, such as competence or strategic behavior.

Finally, it is important to note that in our study we analyze multiple elections: 2012 and 2016. While we find statistically significant results for the reassessment process following the 2012 election, we do not find significant results for the reassessment process following the 2016 election. This suggests that compensating differentials, if present, are not consistently driving the observed outcomes across varying settings.

#### Bandwidth selection.

In any RD model it is important to study whether the results are sensitive to bandwidth selection. Table 10 presents the results of this sensitivity analyses. For each reassessment process we start with the optimal bandwidth (highlighted in bold in each panel), and analyze whether the results change if we modify the bandwidth by a factor of ±10%, ±20%, and ±30%. The results confirm that our findings remain consistent in terms of direction and statistical significance across a range of bandwidths. The effect for the 2014 process remains statistically significant for all bandwidth that we tested, while the effect for the 2018 process is not statistically significant in all specifications. This provides further confidence in the robustness of our results.

**Table 10:** Sensitivity of main results to bandwidth selection

**Panel A: 2014 reassessment**				
**Factor**	**Bw**	**Coefficient**	**St error**	**Robust p-value**
0.7	0.082	1.347	0.251	0.000
0.8	0.094	1.232	0.25	0.000
0.9	0.105	1.182	0.248	0.000
**Optimal**	**0.117**	**1.121**	**0.244**	**0.000**
1.1	0.129	0.991	0.247	0.000
1.2	0.14	0.863	0.253	0.000
1.3	0.152	0.785	0.259	0.000
**Panel B: 2018 reassessment**				
**Factor**	**Bw**	**Coefficient**	**St error**	**Robust p-value**
0.7	0.137	-0.299	0.59	0.869
0.8	0.157	-0.386	0.596	0.865
0.9	0.176	-0.467	0.575	0.965
**Optimal**	**0.196**	**-0.482**	**0.521**	**0.509**
1.1	0.216	-0.524	0.485	0.808
1.2	0.235	-0.555	0.46	0.745
1.3	0.255	-0.58	0.44	0.688

*Note*: This table shows the RD coefficients estimated using a triangular kernel for different bandwidth specifications. Optimal corresponds to the optimal bandwidth selected by the estimation, and the different columns show the estimation results when the bandwidth is increased and decreased by a factor of 10%, 20%, and 30%.

#### Alternative empirical specifications.

It is also important to study whether the results of an RD estimation are sensitive to different kernel choices. The analyses can be found in S1 Appendix. Using an Epanechnikov and a triangular kernel, the findings in Table A.7 show we maintain significance in all specifications in 2014. A similar situation occurs when we use a uniform kernel, as in Table A.8, where coefficients remain significant.

Another relevant analysis is the order of the polynomial used to estimate the RD equation. As already mentioned, we obtain our main results by estimating a linear equation on both sides of the cut-off. However, the results remain robust if we use a quadratic polynomial, see Table A.8 and Figure A.3 presented in the online appendix.

#### Controlling for observable characteristics at the municipal level.

To avoid results being driven by differences in observable characteristics near the cut-off point, we include controls for land and building size, and the logarithm of the average municipal income. Given the quasi-experimental behavior of the municipalities near the cut-off point, i.e. where the allocation is theoretically random, *a priori* we should not find very drastic changes in the results. As Table 11 shows, this is indeed the case, and our main results hold.

**Table 11:** Regression discontinuity results (including controls)

**Panel A: 2014 reassessment**				
	**Δ Appraisal**	**Bw**	**Eff. N**	
			**Left**	**Right**
Right margin	0.853***	0.108	454,197	594,641
	(0.250)			
Left margin	-0.723***	0.097	653,355	517,960
	(0.240)			
Restricted sample margin	0.844***	0.117	463,716	654,866
	(0.249)			
**Panel B: 2018 reassessment**				
	**Δ Appraisal**	**Bw**	**Eff. N**	
			**Left**	**Right**
Right margin	0.076	0.209	881,748	1,294,641
	(0.270)			
Left margin	-0.076	0.234	1,279,853	911,770
	(0.281)			
Restricted sample margin	-0.195	0.196	672,325	779,848
	(0.397)			

*Note*: This table shows RD estimates for changes in appraisals for each reassessment process. Each observation consists of a residential property and the dependent variable corresponds to the percentage change in its appraisal after each reassessment process normalized by the standard deviation of these changes in each process. *Effective N* corresponds to the number of observations that fall inside the optimal bandwidth. The restricted sample includes only observations in which the election was decided between a right-wing and left-wing candidate. The RD coefficients are estimated using a triangular kernel. Robust standard errors are clustered at the municipality level. Significance levels: * *p*-value < .1, ** *p*-value < .05, *** *p*-value < .01.

#### Falsification tests.

Another way to test the model’s performance is to see the effect on those variables that should not change during the time frame of the reassessment processes. To do so, we test the impact of election results on income changes at the municipal level from one semester to another. Table 12 shows that there are no significant effects in this variable for either reassessment processes. These results provide further evidence in support of the random assignment assumption behind our identification strategy.

**Table 12:** Falsification test: Effects on income

**Panel A: 2014 reassessment**				
	**Δ Income**	**Bw**	**Eff. N**	
			**Left**	**Right**
Right margin	-0.003	0.134	733,844	732,231
	(0.010)			
Left margin	-0.000	0.149	893,171	736,720
	(0.010)			
Restricted sample margin	-0.000	0.137	537,587	722,689
	(0.011)			
**Panel B: 2018 reassessment**				
	**Δ Income**	**Bw**	**Eff. N**	
			**Left**	**Right**
Right margin	0.010	0.147	677,295	997,337
	(0.007)			
Left margin	-0.007	0.116	744,380	555,749
	(0.006)			
Restricted sample margin	0.007	0.150	492,877	750,494
	(0.008)			

This table shows RD estimates for changes in the average municipal income. Each observation consists of a residential property. *Effective N* corresponds to the number of observations that are inside the optimal bandwidth. The restricted sample includes only observations in which the election was decided between a right-wing and a left-wing mayor. The RD coefficients are estimated using a triangular kernel. Errors are corrected at the municipality level. Significance levels: * *p*-value < .1, ** *p*-value < .05, *** *p*-value < .01.

## 5 Discussion

In this section, we explore and present evidence for several potential explanations of our results. Based on the literature, we identify three main hypotheses that may account for the findings in this paper. First, the “forbearance hypothesis" suggests that changes in property assessments may result from mayors influencing individual compliance with regulations. Second, the “commercial prices hypothesis" posits that incoming mayors implement policies that lead to an increase in commercial property values, which, in turn, drives higher property assessments. Third, it is possible that, for political reasons, mayors exert pressure on the central government to increase tax collections, thereby securing a larger municipal budget. Finally, at the end of the section, we also discuss the economic significance of our estimates.

### 5.1 Forbearance hypothesis

As discussed in Sect 2, the “forbearance hypothesis" suggests that mayors may influence property assessments by affecting individual compliance with property regulations. Forbearance, as defined by [28], refers to the deliberate non-enforcement of laws, often used by politicians to maximize political benefits or maintain voter support. In this context, mayors could strategically allow informal property expansions or renovations to persist without legal regularization, indirectly influencing property values and assessments. This hypothesis provides a compelling framework to understand how local political dynamics may interact with broader tax policies.

If our results are related to this hypothesis, one could expect changes in observable characteristics that are used to determine the property assessments. One of the most important variables is the constructed surface. Table 13 presents the results of estimating our main RD specification using each property’s change in constructed surface as the dependent variable, and we find no differences in either reassessment process. Thus, we conclude that the increase in property assessments cannot be explained by the forbearance hypothesis.

**Table 13:** Forbearance hypothesis Effects on constructed surface

**Panel A: 2014 reassessment**				
	**Δ Const. Surface**	**Bw**	**Eff. N**	
			**Left**	**Right**
Right margin	4.7e-05	0.108	469,844	604,581
	(0.00029)			
Left margin	-4.4e-05	0.097	452,558	521,225
	(0.00029)			
Restricted sample margin	0.00011	0.117	477,445	665,524
	(0.00029)			
**Panel B: 2018 reassessment**				
	**Δ Const. Surface**	**Bw**	**Eff. N**	
			**Left**	**Right**
Right margin	-0.00015	0.209	905,199	1,314,461
	(0.00035)			
Left margin	-0.00026	0.234	929,850	1,411,443
	(0.00036)			
Restricted sample margin	-2.5e-05	0.196	692,949	795,276
	(0.00037)			

This table shows RD estimates for changes in the constructed surface of each property. Each observation consists of a residential property. *Effective N* corresponds to the number of observations that are inside the optimal bandwidth. The restricted sample includes only observations in which the election was decided between a right-wing and a left-wing mayor. The RD coefficients are estimated using a triangular kernel. Errors are corrected at the municipality level. Significance levels: * *p*-value < .1, ** *p*-value < .05, *** *p*-value < .01.

### 5.2 Commercial prices hypothesis

As discussed previously, our results could be associated with changes in commercial property prices driven by other policies. If the assessment model captures these changes, then our results could be explained in part by this channel. To examine this hypothesis, we collected property-level commercial prices from administrative records provided by the SII. This dataset captures all property transactions that occurred in Chile within two years before and after each election.

Considering that there could be differences in the type of properties, we use a hedonic model for property prices. Specifically we estimate a regression where the dependent variable is the commercial price and the explanatory variables include year fixed effects, municipality fixed effects, constructed surface, a categorical variable for the construction material, a categorical variable for construction quality according to SII, and the number of floors in the building and the construction age.

Then we use the residual of this model as an additional control variable in our main specifications. If our results are driven solely by changes in commercial prices, we would expect that the estimated coefficients become insignificant. Table 14 shows that this is not the case. The coefficients for the 2014 reassessment process remain economically and statistically significant. It is interesting to note that there is a slight decrease in the coefficients. For example, in our preferred specification (restricted margin) for 2014, the original coefficient in Table 8 was 1.140 and it decreases to 0.973 when we control for commercial prices. Thus we conclude that commercial prices could be playing a role in explaining part of the results, but most of the effect remains unexplained.

**Table 14:** Regression discontinuity results controlling for commercial prices

**Panel A: 2014 reassessment**				
	**Δ Appraisal**	**Bw**	**Eff. N**	
			**Left**	**Right**
Right margin	0.983***	0.128	670,383	707,647
	(0.241)			
Left margin	-1.111***	0.089	625,654	520,170
	(0.251)			
Restricted sample margin	0.973***	0.131	523,089	694,366
	(0.246)			
**Panel B: 2018 reassessment**				
	**Δ Appraisal**	**Bw**	**Eff. N**	
			**Left**	**Right**
Right margin	0.014	0.207	889,357	1,298,895
	(0.331)			
Left margin	0.164	0.234	1,285,430	922,385
	(0.359)			
Restricted sample margin	-0.441	0.194	678,951	783,489
	(0.512)			

*Note*: This table shows RD estimates for changes in appraisals for each reassessment process using aggregate commercial prices as a control. Each observation consists of a residential property and the dependent variable corresponds to the percentage change in its appraisal after each reassessment process normalized by the standard deviation of these changes in each process. *Effective N* corresponds to the number of observations that fall inside the optimal bandwidth. The restricted sample includes only observations in which the election was decided between a right-wing and left-wing candidate. The RD coefficients are estimated using a triangular kernel. Robust standard errors are clustered at the municipality level. Significance levels: * *p*-value < .1, ** *p*-value < .05, *** *p*-value < .01.

### 5.3 Political influence and incumbent analysis

Having discarded the forbearance and commercial prices hypotheses, it is plausible to conclude that the effects are related to political influence. Ir order to provide further evidence on this hypothesis we conduct an analysis based on the coalition of the incumbent mayor. Specifically, we restrict the sample to focus on elections where one would expect the effects to be more pronounced. This involves re-estimating our main RD model but limiting the analysis to municipalities where a change in coalition occurred. For instance, we exclude cases where the outgoing mayor was from a right-wing coalition, and the incoming mayor is also right-wing. Instead, we focus exclusively on municipalities where a coalition change took place, such as when the outgoing mayor was right-wing and the incoming mayor is either left-wing or independent.

Table 15 presents the results. Compared to our main findings in Table 8, all coefficients increase in magnitude. Thus, the evidence provided in this table aligns with the hypothesis that political partisanship plays a role in property reassessments and local tax decisions.

**Table 15:** Incumbent analysis

**Panel A: 2014 reassessment**				
	**Δ Appraisal**	**Bw**	**Eff. N**	
			**Left**	**Right**
Right margin	1.534***	0.106	350,836	220,390
	(0.317)			
Left margin	-1.307***	0.085	286,224	406,073
	(0.412)			
Restricted sample margin	1.544***	0.122	346,169	209,469
	(0.280)			
**Panel B: 2018 reassessment**				
	**Δ Appraisal**	**Bw**	**Eff. N**	
			**Left**	**Right**
Right margin	-0.333	0.092	256,121	316,228
	(0.591)			
Left margin	-0.300	0.139	567,458	319,284
	(0.573)			
Restricted sample margin	-0.270	0.129	253,508	419,066
	(0.585)			

This table shows RD estimates restricting the sample to municipalities where the incoming mayor is different that the outgoing mayor. Each observation consists of a residential property. *Effective N* corresponds to the number of observations that are inside the optimal bandwidth. The restricted sample includes only observations in which the election was decided between a right-wing and a left-wing mayor. The RD coefficients are estimated using a triangular kernel. Errors are corrected at the municipality level. Significance levels: * *p*-value < .1, ** *p*-value < .05, *** *p*-value < .01.

Finally, the fact that our main results show significant findings for right-wing mayors only when the main government is led by a right-wing coalition provides additional evidence supporting the political partisanship channel. Although it is not possible for us to provide empirical evidence on the specific mechanism through which local mayors influence the reassessment process - given that such actions would be illegal - the evidence strongly suggests that political partisanship is indeed playing a role in this process.

In general, it is difficult to pinpoint only one specific channel. As it is usually the case in economics many forces are acting at once. However, our evidence suggest that commercial prices and enforcement are not explaining the increase in valuation and thus taxes paid by individuals. Our results suggest that there is an effect of political partisanship on local taxes.

Following [43], we exercise caution in attributing the entirety of the observed effect to a mayor belonging to the right-wing coalition. It is possible that compensating differentials, correlated with the mayor’s coalition but unobservable to us, could also play a role. Therefore, we interpret our results with caution, acknowledging that they may partially reflect unobservable characteristics of the politicians themselves.

### 5.4 Relevance and heterogeneity of results

The previous sections have shown that there is sufficient evidence that local political factors are associated with changes in residential property tax appraisals in Chilean municipalities. In our preferred specification, the changes are economically significant, representing an increase of 31% in the 2014 process. For the average house in our sample, this corresponds to an increase in the appraisal of around $7,000 US$ for 2014. This is a significant amount considering the GDP per capita in Chile in 2014 was $16,675 US$ [44].

An important point to consider is that the SII is not an autonomous body, it is run by the Ministry of Finance. Thus, the national political coalition in power could be influencing appraisals to benefit those municipalities that share their political ideology. For example, [45] show that in Brazil the federal government makes fewer discretionary transfers to municipalities with a mayor from the opposing coalition. In Chile a right-wing coalition was in power during the 2014 reassessment process; in 2014, it was a left-wing coalition. Hence, this is suggestive evidence that political alignment between central and local governments could play a role on local taxes. More evidence on this hypothesis could represent an interesting topic for future research. An alternative explanation for the difference between the 2014 and 2018 results is that the property reassessments have become more salient in recent years for the general population and there have been many proposals by different political sectors to modify this tax in order to reduce the burden for voters.

In terms of the political motivation to influence the assessments, it is interesting to analyze if there is a differential effect for properties that are subject to or exempt from paying property taxes. As previously explained, properties with assessments below a specific value are exempt from paying property taxes. We explore this question in the S1 Appendix where we show that there are no apparent differences in the results if we split the sample between properties that are subject to and exempt from property taxes.

## 6 Conclusion

The evidence presented in this paper demonstrates that political partisanship significantly influences property reassessments and property taxes. Using property-level data from Chile and a regression discontinuity design based on close elections, we address potential omitted variables driving the observed positive association between partisanship and residential property assessments. In our preferred specification, the election of a right-wing mayor over a left-wing one increases assessments by up to 31%, representing a one standard deviation increase on average. This increase implies higher property taxes for certain property owners, enabling greater municipal spending by mayors.

This study contributes to the literature by providing empirical evidence on the impact of political partisanship on local property tax reassessments in an emerging economy. This finding is particularly notable, as previous research has often focused on developed economies, where institutional constraints limit local political influence on tax policy. Our results stand in contrast to much of the existing literature, which finds either no effect (e.g., [11,13]) or that left-wing governments are associated with higher taxes (e.g., [12,15]).

We acknowledge, following [43], that the observed effects may partially reflect compensating differentials. Thus, our findings should be interpreted with caution, as they could represent a combination of political partisanship and unobservable individual traits. However, robustness tests using observable characteristics, such as age and experience, suggest that these factors are unlikely to be fundamental drivers of our results.

Additionally, we tested both the forbearance and commercial prices hypotheses as potential mechanisms. While commercial prices may account for part of the observed effect, neither hypothesis seems sufficient to fully explain our findings. Our analysis also includes an incumbent analysis, which reveals that the observed effects are stronger when the sample is restricted to municipalities where the incoming mayor represents a change in political coalition. These results further support the conclusion that political partisanship plays a role in shaping property reassessments and local tax policies.

Future research could explore the long-term consequences of these changes. On one hand, higher property taxes may result in voter backlash, reducing re-election chances. On the other, increased municipal budgets could enhance public spending, potentially improving re-election prospects. Additionally, further studies could investigate the broader institutional implications of allowing ostensibly non-partisan tax reassessment processes to be influenced by local political factors.

## Supporting information

S1 Appendix (PDF)
