# Supplementary material for: Do political parties matter for property taxes?
Source: PLoS One. 2025 May 22;20(5):e0319994. doi: 10.1371/journal.pone.0319994 (PMC12097557; doi:10.1371/journal.pone.0319994)
Supplement: S1 Appendix — (PDF) [file pone.0319994.s001.pdf]

ONLINE APPENDIX

Do political parties matter for municipal finances?

List of Figures

A.1 Density test: 2014 reassessment . . . iii
A.2 Density test: 2018 reassessment . . . iv
A.3 Regression discontinuity (quadratic polynomial) . . . v
A.4 Regression discontinuity constructed surface . . . vi

List of Tables

A.1 Variable Description . . . vii
A.2 OLS estimates for appraisal changes . . . viii
A.3 Pooled OLS estimates for appraisal changes . . . ix
A.4 Municipalities included in RDD analysis - 2014 (right margin) . . . x
A.5 Municipalities included in RDD analysis - 2018 (right margin) . . . xi
A.6 Regression discontinuity results - Optimal bandwidth from pooled RD . . . xii
A.7 Regression discontinuity results (Epanechnikov kernel) . . . xiii
A.8 Regression discontinuity results (Uniform kernel) . . . xiv
A.9 Regression discontinuity results (quadratic polynomial) . . . xv
A.10 Regression discontinuity results according to payment of property tax: 2014 reassessment . . . xvi
A.11 Regression discontinuity results according to payment of property tax: 2018 reassessment . . . xvii

A.12 Regression discontinuity results using residual of commercial prices before elections xviii

A Figures and Tables

Fig A.1. Density test: 2014 reassessment

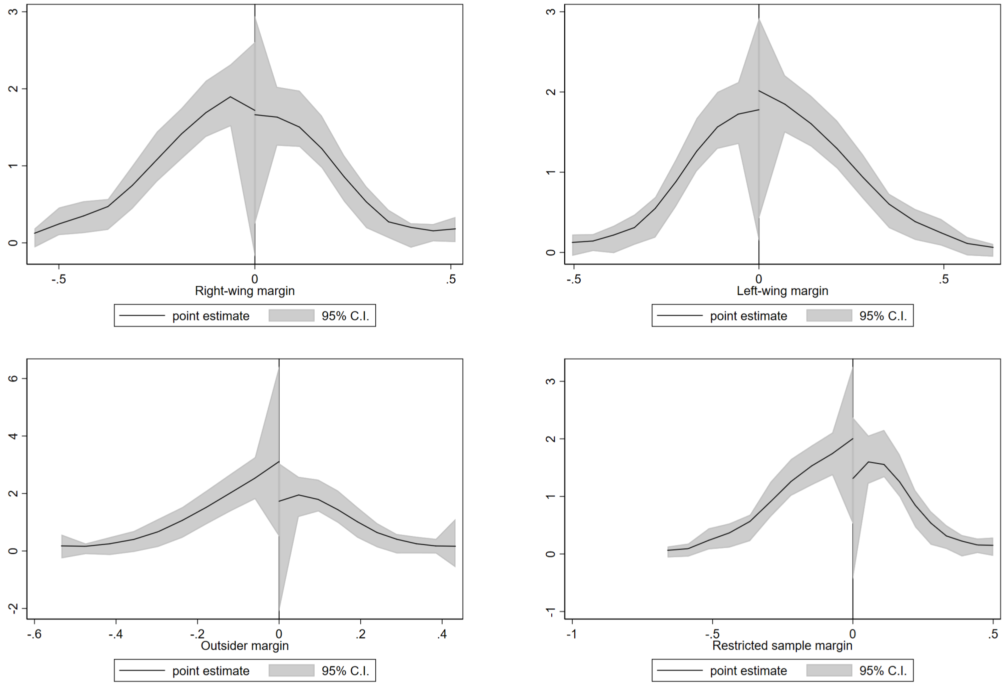

Note: This figure shows density estimators for the RD running variable, which is the margin obtained by the mayor of each coalition for the 2012 municipal election. A significant difference in the density near the cutoff would provide evidence of coalitions having a systematic impact on election results.

Fig A.2. Density test: 2018 reassessment

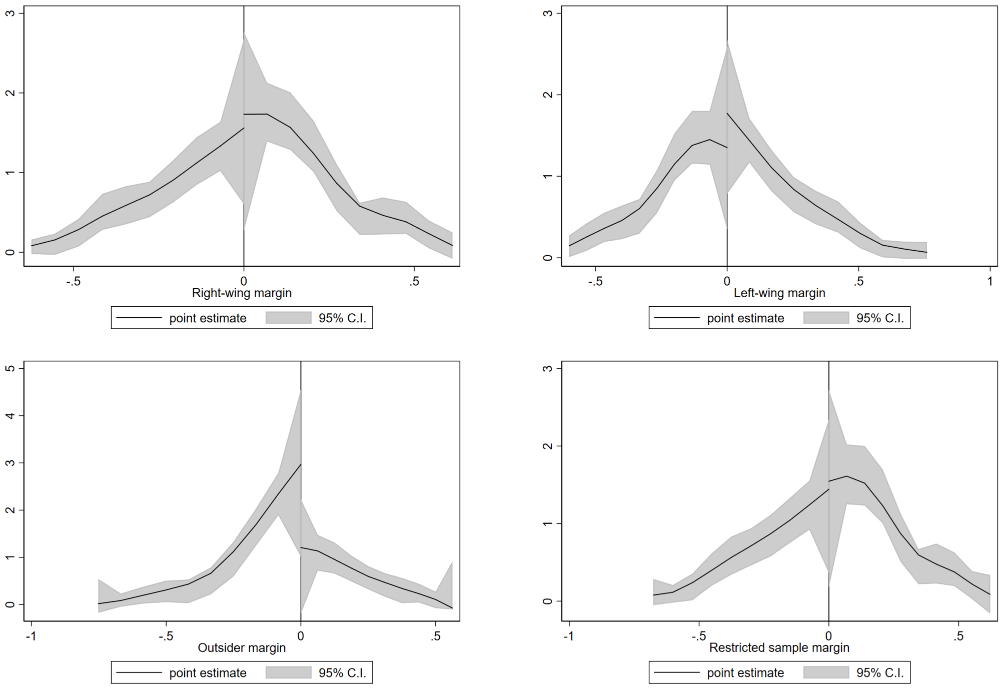

Note: This figure shows density estimators for the RD running variable, which is the margin obtained by the mayor of each coalition for the 2016 municipal election. A significant difference in the density near the cutoff would provide evidence of coalitions having a systematic impact on election results.

Fig A.3. Regression discontinuity (quadratic polynomial)

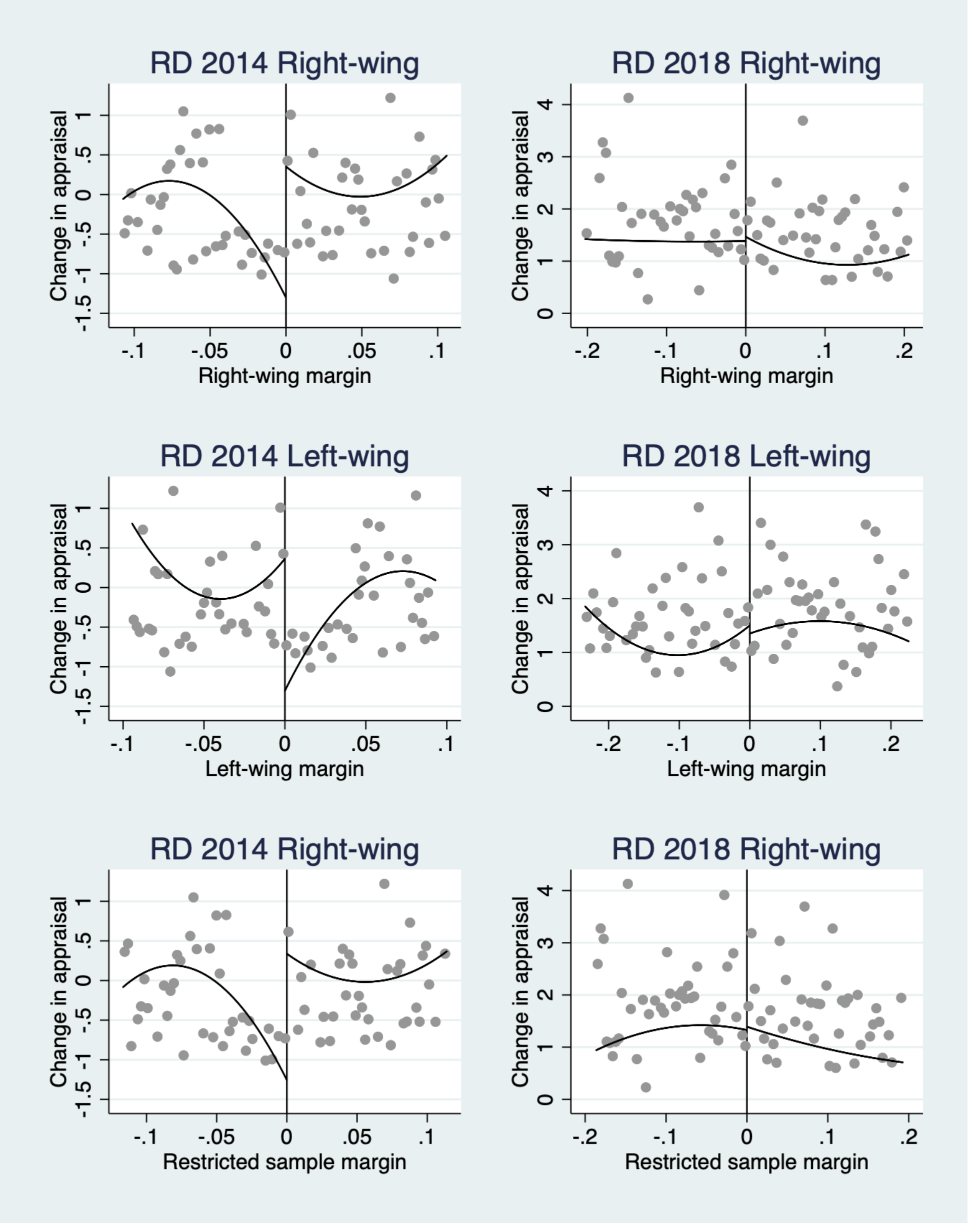

Note: This figure shows regression discontinuity results for close elections (both 2012 and 2016 municipal elections) and their subsequent effect on appraisals. The running variable is the margin between the top two candidates in the election, so falling on the right side of the cutoff means that the right-wing or the left-wing candidate was elected. The bottom row includes only observations in which the election was decided between left-wing and right-wing candidates (i.e., the restricted sample). In this case, falling to the right (left) of the cutoff means the right-wing (left-wing) candidate was elected, while falling on the left means the left-wing candidate was elected. Results were estimated using a quadratic polynomial.

Fig A.4. Regression discontinuity constructed surface

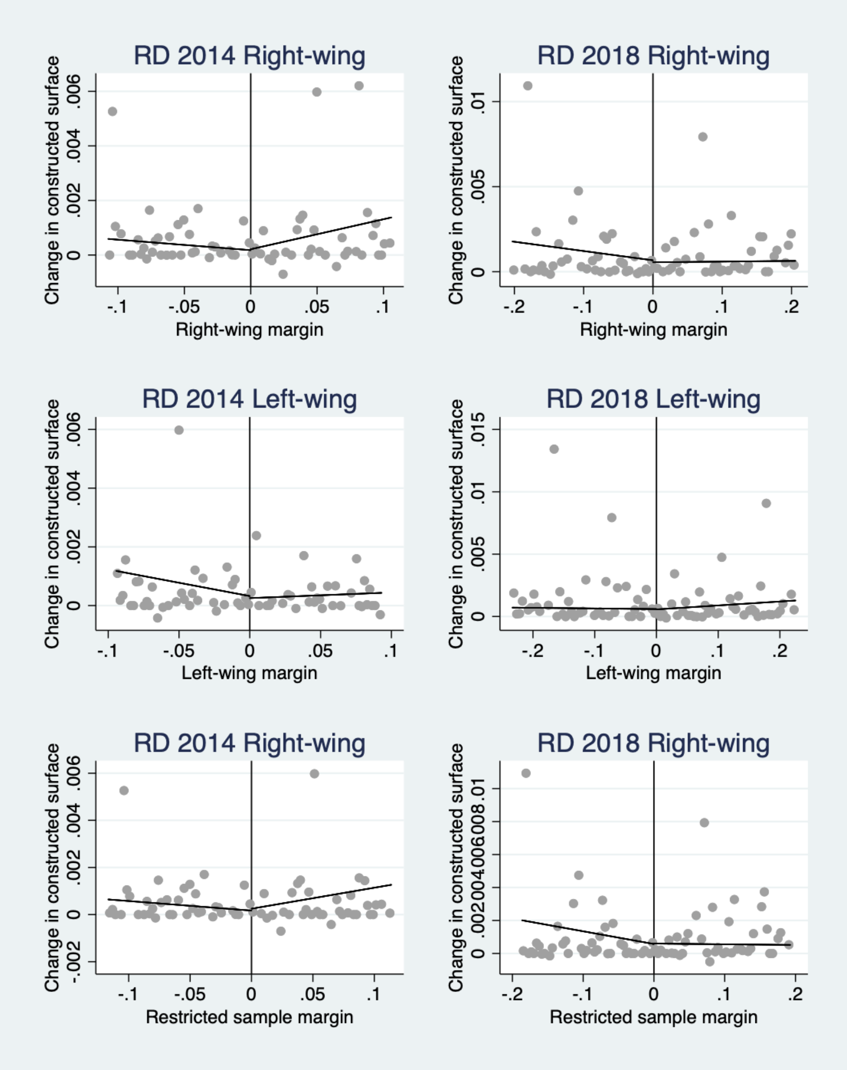

Note: This figure shows regression discontinuity results for close elections (both 2012 and 2016 municipal elections) and their subsequent effect on constructed surface. The running variable is the margin between the top two candidates in the election, so falling on the right side of the cutoff means that the right-wing or the left-wing candidate was elected. The bottom row includes only observations in which the election was decided between left-wing and right-wing candidates (i.e., the restricted sample). In this case, falling to the right (left) of the cutoff means the right-wing (left-wing) candidate was elected, while falling on the left means the left-wing candidate was elected. Results were estimated using a quadratic polynomial.

Table A.1. Variable Description

Variable	Definition	Source
**Fiscal valuation variables**		
$\Delta Valuation$	Percent change in fiscal valuation of the property due to reassessment.	SII
**Elections and income variables**		
Right margin	Voting margin obtained by the right-wing candidate in a mayoral election, considering only the top two candidates.	SERVEL
Left margin	Voting margin obtained by the left-wing candidate in a mayoral election, considering only the top two candidates.	SERVEL
Independent margin	Voting margin obtained by a candidate who doesn't belong to either a right-wing or left-wing coalition in a mayoral election, considering only the top two candidates.	SERVEL
Restricted sample margin	Voting margin obtained by a candidate in an election where the top two candidates were members of both a right-wing and a left-wing coalition. A margin greater than 0 corresponds to a mayor of the right being elected, while a negative value means that the candidate from the left was elected.	SERVEL
Average municipal income	Average income earned by the municipality's residents.	SP
**Additional variables**		
Total general constructions surface	Sum of the surfaces of all constructions inside the municipality classified as general constructions (primarily destined to be habitable). Measured in square meters.	SII
Number of general constructions	Total number of constructions inside the municipality classified as general constructions (primarily destined to be habitable).	SII
Number of non-habitable constructions	Total number of constructions inside the municipality classified as non-habitable constructions.	SII

Note: This table presents definitions and sources for the variables used in this study. SII corresponds to the Chilean Internal Revenue Service (*Servicio de Impuestos Internos*). SERVEL corresponds to Chilean Electoral Service (*Servicio Electoral*). SP corresponds to Chilean Superintendency of Pensions (*Superintendencia de Pensiones*).

Table A.2. OLS estimates for appraisal changes

	2014			2018		
Variables	(1)	(2)	(3)	(4)	(5)	(6)
Right	-0.023***	-0.019***	0.002***	-0.057***	-0.024***	0.013***
	(0.000)	(0.001)	(0.001)	(0.001)	(0.002)	(0.001)
Left	-0.043***	-0.038***	-0.000	0.098***	0.076***	0.037***
	(0.000)	(0.001)	(0.000)	(0.001)	(0.001)	(0.001)
ln(Income)			0.258***			-0.516***
			(0.003)			(0.008)
Constant	0.070***	0.052***	-3.359***	0.672***	-0.133***	6.732***
	(0.000)	(0.001)	(0.046)	(0.001)	(0.001)	(0.111)
Controls						
Land area	No	Yes	Yes	No	Yes	Yes
Material/Quality	No	Yes	Yes	No	Yes	Yes
Special condition	No	Yes	Yes	No	Yes	Yes
Construction age	No	Yes	Yes	No	Yes	Yes
Shared Amenities	No	Yes	Yes	No	Yes	Yes
N	4,332,542	4,332,542	4,332,542	4,747,412	4,747,412	4,747,412

This table shows OLS estimates for changes in appraisals for each reassessment process. Each observation consists of a residential property and the dependent variable corresponds to the percentage change in its appraisal after each reassessment process. The *Income* variable corresponds to the municipal average. Robust standard errors in parentheses. Significance levels: * p-value < .1, ** p-value < .05, *** p-value < .01.

Table A.3. Pooled OLS estimates for appraisal changes

	2014 & 2018 pooled		
Variables	(1)	(2)	(3)
Right	-0.046***	-0.037***	-0.037***
	(0.000)	(0.000)	(0.000)
Left	0.024***	0.012***	-0.002
	(0.000)	(0.000)	(0.000)
ln(Income)			-0.120***
			(0.001)
Controls			
Year f.e	Yes	Yes	Yes
Land area	No	Yes	Yes
Material/Quality	No	Yes	Yes
Special condition	No	Yes	Yes
Construction age	No	Yes	Yes
Shared Amenities	No	Yes	Yes
N	9,079,954	9,079,954	9,079,954

Note: This table shows OLS estimates for changes in appraisals for both reassessment processes combined. Each observation consists of a residential property and the dependent variable corresponds to the percentage change in its appraisal after each reassessment process. The *Income* variable corresponds to the municipal average. Standard errors in parentheses. Significance levels: * p-value < .1, ** p-value < .05, *** p-value < .01.

Table A.4. Municipalities included in RDD analysis - 2014 (right margin)

Municipality	Properties	Margin	Municipality	Properties	Margin
AISEN	5931	6.98%	LONGAVI	3403	-1.31%
ALTO DEL CARMEN	852	0.82%	LOS ALAMOS	3661	3.7%
ANCUD	8090	-5.47%	LOS ANGELES	43209	-8.26%
ANGOL	14937	-2.64%	LOS SAUCES	1552	1.65%
CABILDO	4666	-10.73%	MARIQUINA	3349	-10.33%
CALBUCO	2728	-8.84%	MAULLIN	2282	-1.14%
CALDERA	6764	9.29%	MELIPEUCO	871	-6.43%
CASABLANCA	6688	1.06%	MONTE PATRIA	9677	-8.4%
CANETE	5241	-10.11%	MULCHEN	6590	-2.35%
CERRILLOS	21160	-2.76%	NINHUE	583	6.48%
CERRO NAVIA	29660	3.39%	NUEVA IMPERIAL	5775	-2.97%
CHILLAN	51572	3.91%	OLIVAR	2191	10.12%
CHONCHI	1362	-7.82%	OLMUE	4483	7.9%
CISNES	1322	-7.87%	PADRE LAS CASAS	13961	9.44%
COBQUECURA	990	1.72%	PALENA	349	7.38%
COCHAMO	283	-4.7%	PALMILLA	2353	-0.14%
COIHUECO	3544	2.56%	PANGUIPULLI	5206	1.76%
COINCO	1132	10.55%	PANQUEHUE	1389	10.59%
COLCHANE	199	10.52%	PARRAL	10642	7.99%
CONCON	14704	-6.89%	PEMUCO	1604	9.87%
CONSTITUCION	11542	5.03%	PENCAHUE	1314	8.09%
CONTULMO	807	-2.66%	PENCO	9741	-4.35%
CORONEL	31103	3.8%	PERALILLO	2427	-8.45%
CORRAL	1438	8.3%	PICA	1446	-2.86%
CUNCO	3139	-4.65%	PLACILLA	1490	-0.49%
CURANILAHUE	6751	9.34%	PUERTO MONTT	59828	-7.58%
DALCAHUE	1177	9.85%	PUERTO OCTAY	1075	-7.81%
EMPEDRADO	802	1.62%	PUMANQUE	484	4.24%
ERCILLA	1327	3%	PUREN	2628	-1.39%
ESTACION CENTRAL	32486	1.3%	PURRANQUE	4432	-3.17%
FLORIDA	1575	-5.01%	QUEILEN	567	9.72%
FRESIA	2274	7.11%	QUELLON	2644	-5.95%
FUTALEUFU	357	-9.23%	QUILLECO	1809	9.31%
GORBEA	3355	-1.54%	QUILLON	3786	9.12%
HUALANE	2251	3.99%	RANQUIL	881	5.69%
HUALPEN	27242	-6.02%	RINCONADA	2448	-1.27%
JUAN FERNANDEZ	177	-7.48%	RIO CLARO	2238	0.7%
LA CRUZ	4828	5.27%	SAN GREGORIO	109	-5.21%
LA ESTRELLA	690	8.46%	SAN JAVIER	11809	-4.16%
LA FLORIDA	104510	7.25%	SAN JOSE DE MAIPO	3450	8.76%
LA HIGUERA	2070	3.12%	SAN NICOLAS	1377	-0.64%
LA LIGUA	9734	-7.95%	SAN PEDRO	701	-1.13%
LA UNION	7561	-8.52%	SANTA BARBARA	2585	-4.52%
LAGO RANCO	1089	-4.7%	SANTIAGO	89227	-6.42%
LAGO VERDE	227	4.86%	TEODORO SCHMIDT	2172	-7.24%
LAJA	5582	-3.89%	TIERRA AMARILLA	2628	-10.21%
LAMPA	16828	4.54%	TIMAUKEL	16	5.72%
LAS CABRAS	5785	4.77%	TIRUA	1180	-6.67%
LICANTEN	1833	-9.83%	TOCOPILLA	7959	-7.49%
LINARES	26584	0.76%	VALDIVIA	39122	0.22%
LITUECHE	1199	2.42%	VALPARAISO	77266	10.16%
LLANQUIHUE	3762	-8.14%	NUNOA	78176	0.04%

Table A.5. Municipalities included in RDD analysis - 2018 (right margin)

Municipality	Properties	Margin	Municipality	Properties	Margin	Municipality	Properties	Margin
ALTO HOSPICIO	25743	-9.62%	JUAN FERNANDEZ	185	2.26%	PLACILLA	1687	-20.29%
ANCUD	9810	-16.6%	LA CRUZ	6432	10.12%	PORTEZUELO	690	15.45%
ANDACOLLO	3539	-17.24%	LA ESTRELLA	746	11.55%	PROVIDENCIA	68218	10.84%
ANTOFAGASTA	101201	-5.78%	LA LIGUA	10224	-7.04%	PUCHUNCAVI	9960	-9.54%
BUIN	23948	20.47%	LA PINTANA	46380	-3.59%	PUDAHUEL	61771	-18.41%
CABO DE HORNOS Y ANTARTICA	392	-1.72%	LA REINA	27570	2.09%	PUREN	2927	-18.63%
CALAMA	43004	11.3%	LA UNION	8385	11.46%	PUYEHUE	1839	1.58%
CALERA DE TANGO	4259	-13.5%	LAGO VERDE	251	6.64%	QUEILEN	842	-16.18%
CAMIÃA	419	3.19%	LAJA	5699	3.19%	QUELLON	4160	-2.58%
CARAHUE	4499	4.62%	LANCO	3779	15.78%	QUEMCHI	792	3.48%
CASTRO	10156	8.88%	LAS CABRAS	6517	14.08%	QUILACO	720	7.78%
CATEMU	3756	18.97%	LICANTEN	2132	-16.33%	QUILLON	4699	19.14%
CERRO NAVIA	29458	-2.58%	LINARES	28600	19.73%	QUILPUE	49856	-16.09%
CHAITEN	1031	10.73%	LITUECHE	1367	20.57%	QUINCHAO	935	7.61%
CHANCO	1672	13.52%	LO BARNECHEA	28129	19.7%	QUINTA DE TILCOCO	3096	-8.03%
CHEPICA	4307	12.24%	LO PRADO	23568	-6.78%	QUINTA NORMAL	30077	-10.32%
CHILLAN	58486	19.7%	LONCOCHE	6150	0.16%	QUIRIHUE	3519	-7.4%
CHIMBARONGO	7894	0.78%	LONGAVI	4063	6.08%	RECOLETA	37983	-17.24%
CHOLCHOL	1219	-14.78%	LONQUIMAY	1200	-8.31%	RENGO	16181	-17.31%
CISNES	1519	9.08%	LOS ANDES	21924	10.03%	RETIRO	3184	8.64%
COBQUECURA	1131	-9.7%	LOS LAGOS	3390	-11.43%	RINCONADA	2687	-14.4%
COCHAMO	310	-2.81%	LOS SAUCES	1720	3.09%	RIO BUENO	6657	20.28%
COELEMU	3161	-3.34%	LOS VILOS	8898	-19.92%	RIO CLARO	2645	9.19%
COIHUECO	4109	13.73%	LOTA	12035	-2.46%	RIO HURTADO	1665	9.68%
COINCO	1308	12.57%	MACHALI	17186	3.57%	SAN BERNARDO	79776	20.25%
COLCHANE	207	-16.82%	MAIPU	155529	4.58%	SAN CARLOS	12879	15.85%
COLTAUCO	5316	-14.31%	MALLOA	2705	11.69%	SAN FABIAN	706	-6.32%
CONCEPCION	70871	-12.43%	MARIA ELENA	93	16.7%	SAN FERNANDO	24409	-0.72%
CONCON	17664	-4.49%	MARIA PINTO	2290	-15.45%	SAN IGNACIO	2833	11.66%
CONSTITUCION	13601	17.67%	MARIQUINA	4207	13.38%	SAN JAVIER	13238	-7.81%
CONTULMO	935	11.74%	MEJILLONES	3125	2.84%	SAN JOSE DE MAIPO	3775	13.34%
CORRAL	1515	3.2%	MELIPEUCO	898	11.8%	SAN MIGUEL	39533	2.46%
CUNCO	3272	-8.78%	MONTE PATRIA	9998	-6.49%	SAN NICOLAS	1546	-9.66%
CURACAVI	7466	19.77%	MOSTAZAL	5678	7.65%	SAN PABLO	1732	8.17%
CURACO DE VELEZ	513	-0.09%	NANCAGUA	4916	-13.03%	SAN PEDRO DE ATACAMA	1377	11.42%
CURANILAHUE	7848	2.27%	NEGRETE	1890	-12.29%	SAN VICENTE	12291	-4.24%
CURARREHUE	643	-6.27%	NINHUE	674	0.28%	SANTIAGO	110343	10.1%
CURICO	48219	-10.64%	O'HIGGINS	165	0.7%	TALCA	76472	10.13%
EL MONTE	8534	-18.51%	OLIVAR	2436	-1.2%	TALCAHUANO	41322	3.3%
EL QUISCO	12287	-15.9%	OLLAGUE	74	14.19%	TILTIL	3465	19.89%
EMPEDRADO	904	2.55%	OLMUE	4921	19.26%	TIMAUKEL	13	-16.55%
ERCILLA	1370	6.8%	PAIHUANO	1646	3.81%	TORRES DEL PAINE	13	-1.57%
ESTACION CENTRAL	40990	9.16%	PAILLACO	3641	-7.12%	TUCAPEL	4669	2.21%
FLORIDA	1745	-17.6%	PALMILLA	2733	-17.94%	VALDIVIA	42884	1.97%
FREIRE	2516	7.14%	PANGUIPULLI	5782	-6.86%	VICHUQUEN	1834	15.38%
FRESIA	2624	4.02%	PANQUEHUE	1621	12.24%	VICTORIA	8742	-0.08%
FRUTILLAR	4432	8.79%	PAPUDO	4964	6.87%	VICUÃA	8957	-4.53%
FUTRONO	2552	17.57%	PAREDONES	1376	-2.3%	VILCUN	5675	-17.99%
GENERAL LAGOS	121	-3.9%	PELLUHUE	3136	-16.69%	VILLA ALEGRE	4764	-8.22%
GORBEA	3865	11.36%	PEMUCO	1726	8.96%	VITACURA	31488	17.87%
HUALAÃE	2887	15.86%	PENCAHUE	1494	8.62%	YERBAS BUENAS	2808	-20.06%
HUALPEN	28455	-1.19%	PEÃAFLOR	24277	-5.61%	YUNGAY	3803	-4.62%
IQUIQUE	50680	-0.26%	PICA	1475	16.19%	ZAPALLAR	5009	0.02%
ISLA DE MAIPO	7513	-8.74%	PIRQUE	4289	4.93%	ÃUÃOA	89583	13.24%

Table A.6. Regression discontinuity results - Optimal bandwidth from pooled RD

Panel A: 2014 reassessment

	Δ Appraisal	Bw	Eff. N	
			Left	Right
Right margin	0.757***	0.164	785,502	854,800
	(0.266)			
Left margin	-0.717***	0.128	859,272	635,254
	(0.252)			
Restricted sample margin	0.767***	0.156	577,245	716,798
	(0.261)			

Panel B: 2018 reassessment

	Δ Appraisal	Bw	Eff. N	
			Left	Right
Right margin	-0.013	0.164	728,255	1,005,711
	(0.389)			
Left margin	-0.146	0.128	741,597	612,111
	(0.467)			
Restricted sample margin	-0.383	0.024	476,924	738,823
	(0.596)			

Note: This table shows RD estimates for changes in appraisals for each reassessment process. Each observation consists of a residential property and corresponds to the percentage change in its appraisal. *Effective N* corresponds to the number of observations that fall inside the optimal bandwidth. The restricted sample includes only observations in which the election was decided between a right-wing and left-wing candidate. The RD coefficients are estimated using a triangular kernel. Robust standard errors are clustered at the municipality level. Significance levels: * p-value < .1, ** p-value < .05, *** p-value < .01.

Table A.7. Regression discontinuity results (Epanechnikov kernel)

Panel A: 2014 reassessment

	Δ Appraisal	Bw	Eff. N	
			Left	Right
Right margin	1.079***	0.108	458,291	600,612
	(0.255)			
Left margin	-0.915***	0.097	660,316	523,098
	(0.269)			
Restricted sample margin	1.054***	0.117	467,769	660,338
	(0.253)			

Panel B: 2018 reassessment

	Δ Appraisal	Bw	Eff. N	
			Left	Right
Right margin	-0.084	0.209	889,696	1,299,062
	(0.345)			
Left margin	0.258	0.234	1,285,621	922,385
	(0.350)			
Restricted sample margin	-0.549	0.196	678,951	783,656
	(0.491)			

Note: This table shows RD estimates for changes in appraisals for each reassessment process. Each observation consists of a residential property and the dependent variable corresponds to the percentage change in its appraisal after each reassessment process normalized by the standard deviation of these changes in each process. *Effective N* corresponds to the number of observations that fall inside the optimal bandwidth. The restricted sample includes only observations in which the election was decided between a right-wing and left-wing candidate. The RD coefficients are estimated using a triangular kernel. Robust standard errors are clustered at the municipality level. Significance levels: * p-value < .1, ** p-value < .05, *** p-value < .01.

Table A.8. Regression discontinuity results (Uniform kernel)

Panel A: 2014 reassessment

	Δ Appraisal	Bw	Eff. N	
			Left	Right
Right margin	0.844***	0.108	458,291	600,612
	(0.273)			
Left margin	-0.691***	0.097	660,316	523,098
	(0.282)			
Restricted sample margin	0.623**	0.117	467,769	660,338
	(0.311)			

Panel B: 2018 reassessment

	Δ Appraisal	Bw	Eff. N	
			Left	Right
Right margin	-0.190	0.209	889,696	1,299,062
	(0.350)			
Left margin	0.363	0.234	1,285,621	922,385
	(0.332)			
Restricted sample margin	-0.515	0.196	678,951	783,656
	(0.420)			

Note: This table shows RD estimates for changes in appraisals for each reassessment process. Each observation consists of a residential property and the dependent variable corresponds to the percentage change in its appraisal after each reassessment process normalized by the standard deviation of these changes in each process. *Effective N* corresponds to the number of observations that fall inside the optimal bandwidth. The restricted sample includes only observations in which the election was decided between a right-wing and left-wing candidate. The RD coefficients are estimated using a triangular kernel. Robust standard errors are clustered at the municipality level. Significance levels: * p-value < .1, ** p-value < .05, *** p-value < .01.

Table A.9. Regression discontinuity results (quadratic polynomial)

Panel A: 2014 reassessment

	Δ Appraisal	Bw	Eff. N	
			Left	Right
Right margin	1.655***	0.108	458,291	600,612
	(0.351)			
Left margin	-1.690***	0.097	660,316	523,098
	(0.316)			
Restricted sample margin	1.648***	0.117	467,769	660,338
	(0.338)			

Panel B: 2018 reassessment

	Δ Appraisal	Bw	Eff. N	
			Left	Right
Right margin	0.085	0.209	889,696	1,299,062
	(0.484)			
Left margin	-0.126	0.234	1,285,621	922,385
	(0.560)			
Restricted sample margin	-0.110	0.196	678,951	783,656
	(0.679)			

Note: This table shows RD estimates for changes in appraisals for each reassessment process. Each observation consists of a residential property and the dependent variable corresponds to the percentage change in its appraisal after each reassessment process normalized by the standard deviation of these changes in each process. *Effective N* corresponds to the number of observations that fall inside the optimal bandwidth. The restricted sample includes only observations in which the election was decided between a right-wing and left-wing candidate. The RD coefficients are estimated using a triangular kernel. Robust standard errors are clustered at the municipality level. Significance levels: * p-value < .1, ** p-value < .05, *** p-value < .01.

Table A.10. Regression discontinuity results according to payment of property tax: 2014 reassessment

(a) Properties exempt from taxes

	Δ Appraisal	Bw	Eff. N	
			Left	Right
Right margin	0.400***	0.028	59,323	122,307
	(0.016)			
Left margin	-0.383***	0.022	150,677	19,103
	(0.022)			
Reduced sample margin	0.394***	0.027	40,286	120,798
	(0.018)			

(b) Taxable properties

	Δ Appraisal	Bw	Eff. N	
			Left	Right
Right margin)	0.029*	0.169	219,964	159,994
	(0.017)			
Left margin	0.042	0.039	74,423	2,158
	(0.071)			
Reduced sample margin	0.141***	0.088	108,822	110,320
	(0.025)			

Note: This table shows RD estimates for appraisal changes from the 2014 reassessment process. Each observation is a residential property and corresponds to the percentage change in its appraisal. *Effective N* corresponds to the number of observations that are inside the optimal bandwidth. The restricted sample includes only observations in which the election was decided between a right-wing and left-wing mayor. Panel (a) includes properties that are exempt from property taxes because they fall below the defined appraisal threshold. Panel (b) includes properties that are above that threshold. The RD coefficients are estimated using a triangular kernel. Errors are corrected at the block level. Significance levels: * p-value < .1, ** p-value < .05, *** p-value < .01.

Table A.11. Regression discontinuity results according to payment of property tax: 2018 reassessment

(a) Properties exempt from taxes

	Δ Appraisal	Bw	Eff. N	
			Left	Right
Right margin	0.013	0.064	297,035	280,816
	(0.031)			
Left margin	-0.440***	0.025	147,266	67,951
	(0.038)			
Restricted sample margin	0.092**	0.057	172,776	142,918
	(0.046)			

(b) Taxable properties

	Δ Appraisal	Bw	Eff. N	
			Left	Right
Right margin	0.249***	0.194	142,127	373,877
	(0.022)			
Left margin	-0.282***	0.058	86,964	44,274
	(0.024)			
Reduced sample margin	0.311***	0.071	30,020	72,210
	(0.033)			

Note: This table shows RD estimates for changes in appraisals for the 2018 reassessment process. Each observation consists of a residential property and the dependent variable corresponds to the percentage change in its appraisal after each reassessment process normalized by the standard deviation of these changes in each process. *Effective N* corresponds to the number of observations that are inside the optimal bandwidth. The restricted sample includes only observations in which the election was decided between a right-wing and left-wing mayor. Panel (a) includes properties that are exempt of property taxes because they fall below the defined appraisal threshold. Panel (b) includes properties that are above that threshold. The RD coefficients are estimated using a triangular kernel. Errors are corrected at the block level. Significance levels: * p-value < .1, ** p-value < .05, *** p-value < .01.

Table A.12. Regression discontinuity results using residual of commercial prices before elections

Panel A: 2012 election

	Residual	Bw	Eff. N	
			Left	Right
Right margin	-221.39	0.116	59	53
	(134.04)			
Left margin	187.74	0.113	59	60
	(132.55)			
Restricted sample margin	-233.24	0.118	52	48
	(153.47)			

Panel B: 2016 election

	Residual	Bw	Eff. N	
			Left	Right
Right margin	177.43	0.107	41	51
	(118.37)			
Left margin	106.94	0.158	71	64
	(135.79)			
Restricted sample margin	177.72	0.124	36	51
	(129.36)			

Note: This table shows RD estimates for the residual of commercial prices (residuals) before the 2012 and 2016 elections. Each observation consists of a municipality. We estimate a regression where the dependent variable is the commercial price and the explanatory variables include year fixed effects, municipality fixed effects, constructed surface, a categorical variable for the construction material, a categorical variable for construction quality according to SII, and the number of floors in the building and the construction ag *Effective N* corresponds to the number of observations that fall inside the optimal bandwidth. The restricted sample includes only observations in which the election was decided between a right-wing and left-wing candidate. The RD coefficients are estimated using a triangular kernel. Significance levels: * p-value < .1, ** p-value < .05, *** p-value < .01.

B Explanation of the Appraisal Model

The model values agricultural and non-agricultural real estate differently and takes into account different factors. Since we focus only on residential properties, the relevant model is that of non-agricultural real estate. All Chilean homes fall under this category.

The tax assessment of non-agricultural real estate is determined as follows:

$$Fiscal\,Appraisal = Land\,Appraisal + Construction\,Appraisal + Shared\,Amenities\,Appraisal. \tag{1}$$

- *Fiscal Appraisal* refers to the monetary value that determines the contribution payable by the person associated with the *role*. The role is the individual ID number associated with every Chilean property.
- *Land Appraisal* refers to the monetary value associated with the land on which a structure is built. There does not need to be one linked to the role directly.
- *Construction Appraisal* refers to the sum of the valuation of all the structural components (known as *lines*) that make up the construction directly associated with the property.
- *Shared Amenities Appraisal* refers to the appraised value associated with some lands or constructions that are shared between different properties. This component considers the amount of appraisal that is associated with each role after apportionment between the various units that are linked. In the case of non-agricultural housing, amenities are land, warehouses, or parking lots. There may be more than one shared amenity associated by role.

Different factors determine each of these components:

$$Land\,Appraisal = Area \times Land\,Value\,HA \times Guide\,Coef. \times Corrective\,Coef. \tag{2}$$

Where:

- *Surface* refers to the size of the property associated with the role, measured in m^2.
- *Land Value Homogeneous Area* (HA) refers to the value, measured in \$ m^2, of the HA, corresponding to territorial units defined by the SII, considering the location, the urbanization works, and available equipment. The SII also analyzes the urban planning regulations contained in the regional planning instruments for the municipalities in question. Before 2014, the values were defined by *Zones of Similar Characteristics* (ZSCs), which were larger than the HA, so the number of different values per municipality for the parameter was smaller.
- *Guide Coefficient* refers to an extraordinary adjustment that applies to individual blocks or roles within a municipality.
- *Corrective Coefficient* refers to an adjustment for various characteristics, including:
  - Surface adjustment (CS): Applies based on a range defined by HA.
  - Front-Back ratio adjustment (FF): It is applied based on the ratio between the meters located at the front of the property and the ones located at the back of the property.
  - Height adjustment (CA): Determined at HA level if taller and shorter buildings coexist.
  - Exceptional case adjustments (EC): These apply if a particular property presents a duly supported handicap that justifies a lower value.

Thus, we define the correction coefficient as

$$Corrective\ Coefficient = Min[(FF \times CS), CE] \times AC. \tag{3}$$

While we define the construction as

$$Construction\ Appraisal = \sum_{i=1}^{n} Surface_i \times Construction\ Value_i \times Adjustment\ Coef._i. \tag{4}$$

Since a role can have multiple lines associated with it, we compute the construction value as the sum of each of these segments. The components of the line appraisals are:

- *Surface* refers to the line size, measured in m^2 or m^3, depending on the material.
- *Construction Value* refers to the value according to the type, class, and quality of the material used in the line. It is measured in m^2 or m^3, depending on the material.
- *Adjustment Coefficient* considers adjustment factors for special building conditions, age, municipality, and location in commercial areas.

Finally, we define the appraisal of the shared amenities as:

$$Valuation\ of\ Shared\ Amenities = \sum_{j=1}^{n} Total\ Apraisal\ of\ Shared\ Amenity_j \times \%Apportionment \tag{5}$$

- *Total Shared Amenity Appraisal* refers to the total appraisal value calculated using the corresponding tax appraisal formula.
- *% Apportionment* refers to the percentage of the shared amenity assigned to the role, given by the co-ownership contract.

C Municipal Common Fund (FCM) explanation

The *Fondo Común Municipal* (FCM) serves as the the main source of financing for Chilean municipalities. It is established in Article 122 of the Chilean constitution, which states, "An organic constitutional law will contemplate a solidarity redistribution mechanism of own income among the country's municipalities with the name *Fondo Común Municipal*." To accomplish this, a coefficient was developed to divide the total national fund. The coefficient is set at the municipal level and is determined as follows:

$$FCM\,Coefficient = 0.25 \times IDP + 0.1 \times Poverty\,Index + 0.3 \times Property\,Index + 0.35 \times IPP \quad (6)$$

- *Equal parts index* (IDP) refers to a coefficient that is the same for all the municipalities in the country. This means that 25 % of the FCM is distributed equally among all the municipalities.
- *Poverty Index* considers the number of citizens below the poverty line (based on the national CASEN survey and the population of the municipality) divided by the total national population below the poverty line.
- *Property Index* considers (a) the percentage of properties in the municipality that are exempt from property taxes and (b) the percentage of all exempt properties.
- *Own Permanent Income Index* (IPP) considers the per capita income of the municipality. This income comes from property taxes, circulation permits, municipal patents, cleaning rights, and other municipal rights.
